# Factors Influencing Community Members' Perception of Primary Health Care Services Delivered by Community Health Workers in Rural Areas: A Systematic Review

**DOI:** 10.1111/ajr.70142

**Published:** 2026-01-19

**Authors:** Niaz Ahmed, Maginsh Dahal, Hamish Crocket, Roger Strasser

**Affiliations:** ^1^ Te Huataki Waiora School of Health University of Waikato Hamilton New Zealand; ^2^ Northern Ontario School of Medicine (NOSM) University Sudbury Ontario Canada

**Keywords:** community health workers, community perception, community satisfaction, primary care, primary health care, remote area, rural areas

## Abstract

**Introduction:**

Community Health Workers (CHWs) play an important role in delivering primary health care (PHC) services in rural and remote areas. Community perceptions regarding CHWs impact the acceptance, satisfaction and utilisation of health care services. This subsequently affects overall health outcomes and equity within the system. However, the various factors influencing these perceptions are still dispersed across different studies.

**Objective:**

To identify and synthesise the factors influencing community members' perceptions of PHC services delivered by community health workers in rural areas.

**Design:**

Following the Preferred Reporting Items for Systematic Reviews and Meta‐Analysis (PRISMA) 2020 guidelines, a Systematic Review was conducted.

**Method:**

A thorough search across seven databases for English‐language peer‐reviewed publications from 1990 to 2024 yielded 4996 records. Following screening of titles and abstracts, we reviewed 231 full texts and ultimately included 30 articles for in‐depth appraisal.

**Results:**

Articles from 15 countries identified three interconnected thematic clusters affecting community perception: (1) structural and service delivery cluster, including CHW accessibility, service availability and healthcare quality; (2) trust cluster, such as trust, competency and confidentiality and (3) identity and sociocultural cluster, including CHWs' gender and CHWs from the same community.

**Conclusion:**

Incorporating identified factors into the training curriculum for CHWs, their capacity to meet community expectations and build trust can be enhanced. These insights can be used to align services with community needs. This systematic review identified important factors for improving CHWs' delivery of PHC services in rural areas that ensure these services are sustainable and responsive to community needs.

## Abstract (highlights)

**What Is Already Known:**

- Community perspectives are important in evaluating health care, and the community is more likely to utilise healthcare services if they perceive them as acceptable.
- Perspectives of Community Health Workers regarding challenges and barriers faced in their role of delivering primary health care services.
- There are individual studies on community perceptions and factors affecting community perception regarding primary health care service delivery, but evidence in the form of a systematic review is lacking.

**What Does This Paper Add?:**

- This paper provides an overview of the factors influencing community members' perceptions of primary health care services delivered by community health workers (CHW) in rural areas.
- This review presents the community's viewpoint on the culturally responsive healthcare services, focusing on the quality and effectiveness of these services.
- Healthcare providers can leverage the identified factors to align their services with community needs.
- Most important factors are accessibility to CHWs, trust in CHWs, the availability of services convenient to community members and the gender of CHWs.

## 1 Introduction

Community Health Workers (CHWs) typically deliver services in communities away from established health facilities. They possess a certain level of formal, albeit limited, training for the responsibilities they are meant to fulfil. Their role has evolved over a century as a cornerstone of primary health care (PHC) delivery, particularly in resource‐limited and rural settings. CHW programmes trace their roots back to Ding Xian, China, during the 1920s. During the 1960s, it became increasingly clear that the modern Western medical approach with trained physicians was struggling to meet the needs of rural and impoverished communities across the developing world. This promoted the development of CHW initiatives in multiple countries [1].

In 1978, the Alma‐Ata Declaration, a milestone in global healthcare, firmly committed to the goal of achieving “Health for All” through the promotion of PHC. This approach is the first level of contact, providing accessible healthcare where people live and work. In its Article VII, this declaration highlighted the role of CHWs in delivering PHC [1, 2]. The Alma‐Ata Declaration further emphasised that PHC models should develop in accordance with the nation's economic conditions as well as the socio‐cultural and political traits of its communities. Consequently, countries that signed the Alma‐Ata Declaration viewed the creation of community health worker programmes (CHWP) as integral to the PHC strategy. As a result, during the 1980s, the PHC approach led to large‐scale initiatives for training community health workers across numerous nations [1, 3]. Defining CHWs, however, has remained complex, as each country has developed its own model [4]. According to the World Health Organization (WHO), “Community health workers should be members of the communities where they work, should be selected by the communities, should be answerable to the communities for their activities, should be supported by the health system but not necessarily a part of its organisation, and have shorter training than professional workers” [5, p. 3]. In this way, CHWs are uniquely positioned to manage individual and community health concerns while staying connected to health services [6].

Nevertheless, there is also a degree of role ambiguity for CHWs as they have responsibilities within the health system, but they also collaborate with communities to recognise needs and enhance the involvement and availability of health services [4, 5]. CHWs are typically classified as generalists or specialists. Specialist CHWs focus on up to three disease prevention programmes for a specific population, while generalists are responsible for more than three programmes, with diverse tasks and serving all age groups [7]. They deliver community‐focused health services that promote, prevent and treat illnesses [8].

Community Health Workers (CHWs) can improve access to health care. They deliver maternal and child health (MCH) services. In specific situations, their efforts have been shown to reduce child mortality rates [1, 9, 10, 11, 12, 13]. Evidence also indicates that CHWs can help alleviate the burden on healthcare systems and improve access to essential health services, improving immunisation, especially in remote areas, and reducing health equity disparities [5, 13, 14]. They are now being increasingly acknowledged as valuable providers of services related to noncommunicable diseases (NCDs) [15], such as diabetes [16], hypertension [17], mental health [18] and cancer screening [19]. They are also recognised for their role in digital health [20], environmental health [21], and humanitarian events [22]. This recognition has grown particularly in light of the coronavirus disease 2019 (COVID‐19) pandemic [23]. Although CHWs were previously considered for interventions in developing nations, CHW programmes are now vital elements even in developed countries [1, 8, 24, 25, 26].

While there have been significant improvements in health outcomes over the last few decades, a new reality has emerged. Evolving health requirements, increasing public expectations, and ambitious new health objectives are raising the expectations for health systems to achieve better health results and enhanced social value. What is necessary are high‐quality health systems that optimise healthcare in each specific context, being trusted and valued by everyone, and adapting to the changing needs of the population [27]. Efforts to enhance population health have commonly concentrated solely on increasing access to basic health services, overlooking the importance of care quality [28]. While increasing access to healthcare services remains a global priority, there is growing recognition that access alone does not guarantee improved health outcomes unless the quality of care is also ensured [27, 28]. An increasing awareness exists that individuals might be behaving in a completely rational manner when they choose to refrain from utilising health services that are of low quality. Furthermore, the inadequacy of care can serve as an obstacle to achieving universal health coverage (UHC), regardless of accessibility [28]. Therefore, understanding how recipient communities evaluate service quality is essential, especially for capturing the true impact of CHWPs on health outcomes and service equity.

While CHWs play an increasingly vital role in improving healthcare outcomes, evaluating the quality of services they provide presents unique complexities [29]. In contrast to the quality of goods, which can be objectively assessed using indicators like durability and defect count, the quality of services is a complex and abstract construct. In the absence of objective metrics, a suitable method for evaluating the quality of service is to gauge consumers' perceptions of that service [30]. The quality of service that consumers perceive is derived from comparing their expectations of what the service provider should deliver with their actual experience of the provider's performance [30, 31, 32, 33, 34]. The factors influencing the behavioural (utilisation) and subjective (satisfaction) outcomes of pursuing care might originate from characteristics of the individuals or from the health care system they are attempting to access [35]. Therefore, understanding perception, which is a cognitive appraisal process through which individuals evaluate and interpret health services [36], satisfaction (a subjective outcome) and utilisation (a behavioural outcome) [35], is critical for assessing the effectiveness (a health system performance) [27] of CHW‐led interventions. Health beliefs encompass the attitudes, values and knowledge individuals possess regarding health and healthcare services, which may affect their later perceptions of necessity and utilisation of those services. These beliefs about health offer insight into how social structures can affect enabling resources, perceived needs and the eventual use of healthcare services [37].

Various theoretical models have been developed to examine how consumers perceive and assess service quality in a healthcare setting. These frameworks help us understand patient satisfaction. They also help understand how individuals' expectations and experiences shape perceptions of quality, trust and the use of health services. The Andersen Behavioural Model offers a comprehensive theoretical framework in which “perceptions” (as part of predisposing factors, including beliefs and attitudes) influence individuals' propensity to utilise health services. Satisfaction is considered an outcome of actual service utilisation. It indicates the extent to which experiences of services meet expectations. Utilisation is determined by a combination of predisposing characteristics (such as perceptions), enabling resources and need factors. Ultimately, effective utilisation, shaped by positive perceptions and resulting satisfaction, can contribute to improved individual health outcomes and perceived quality of care [37, 38]. Rosenstock's Health Belief Model (HBM) clarifies how individual perceptions affect health behaviours. In this model, cues to action, like CHW visits or health education from a CHW, trigger these perceptions into actual use of services [39].

In this context, satisfaction is subjective and unique to every individual. It results from the interaction between the perception of the service and the expectations that consumers hold for that service. As a result, different consumers can experience varying degrees of satisfaction from an essentially identical experience [40]. Donabedian's model further conceptualises satisfaction as an outcome of the interaction between healthcare structures (e.g., trained CHWs, resources) and processes (e.g., culturally appropriate, consistent and timely healthcare) [41]. Patient satisfaction has an influence on health status and medical outcomes. Levels of patient satisfaction have been shown to influence rates of utilisation and compliance [42]. Recognising this, the WHO asserts that ensuring public satisfaction with its performance is one of the main goals of a health system [43].

So, understanding community satisfaction is essential in healthcare, particularly regarding the vital role of CHWs, who are members of the communities they serve and have been selected by the communities to be answerable to the communities for their services [44]. In various rural and remote areas, CHWs often serve as the first—and sometimes only—point of contact for health services. The acceptability of CHW‐delivered care directly shapes health‐seeking behaviour and ongoing engagement with the broader health system. Community satisfaction with CHWs' services at this entry level of the system has lasting consequences, influencing not only retention with CHWs but also subsequent utilisation of other health services.

Therefore, understanding client characteristics that influence satisfaction with CHWs is essential for identifying factors linked to positive care experiences and client retention. However, the satisfaction literature shows conflicting findings about factors associated with satisfaction due to the absence of a universal method for measuring satisfaction. Nonetheless, studies indicate that satisfaction varies based on demographics in different healthcare settings [45]. Hence, identifying the factors that affect the perception of the community regarding CHWs' service delivery becomes very important and would help improve acceptance, the utilisation and satisfaction of their services.

While there are numerous studies regarding the perception of community members, there is an evident research gap in comprehensively identifying the factors that shape community members' perceptions of PHC delivery by CHWs, specific to rural areas, through a systematic review. This systematic review aimed to identify factors affecting the perception of community members regarding the service delivery of primary health care services by community health workers in rural areas.

## 2 Methodology

### 2.1 Study Design

Following the Preferred Reporting Items for Systematic Reviews and Meta‐Analysis (PRISMA) guidelines [46], this systematic review was completed.

### 2.2 Search Strategy

A comprehensive search strategy was developed utilising search terms designed to identify relevant studies, incorporating keywords related to community health workers, primary health care, community perceptions and rural areas, as outlined in Table 1 (Supporting Information S1). A detailed compilation of the various names for Community Health Workers employed across different countries was created, based on a systematic review of the existing literature [47, 48]. In the search strategy, we used all terms identified for community‐based practitioners from the systematic review of existing reviews on CHWs [48] to ensure no CHW names were overlooked due to varying names being used worldwide (Supporting Information S1).

**TABLE 1:** Search terms.

Search terms
Community Health Worker, CHW, Accompagnateur, Accredited Social Health Activist, Activista, Agente comunitário de saúde, Anganwadi, Animator, Animatrice, ASHA, Barangay Health Worker, Basic Health Worker, Behvarz, Barefoot Doctor, Bridge‐to‐Health Team, Brigadista, Care Group, Care Group Volunteer, Close‐to‐Community Provider, Colaborador Voluntario. Community‐Based Practitioner, Community Case Management Worker, Community Drug Distributor, Community Health Aide, Community Health Agent, Community Health Care Provider, Community Health Assistant, Community Health Extension Worker, Community Health Distributor, Community Health Officer, Community Health Promoter, Community Health Practitioner, Community Health Representative, Community Health Surveyor, Community Health Volunteer, Community Practitioner, Community Resource Person, Community Surveillance Volunteer, Community Volunteer, Family Health Worker, Family Planning Agent, Family Welfare Assistant, Female Community Health Volunteer, Female Multipurpose Health Worker, Frontline Health Worker, Health Agent, Health Assistant, Health Auxiliary, Health Extension Worker, Health Promoter, Health Surveillance Assistant, Kader, Lay Health Worker, Lady Health Worker, Lead Mother, Malaria Agent, Maternal and Child Health Worker, Mobile Clinic Team, Monitora, Mother Coordinator, Nutrition Agent, Nutrition Counsellor, Outreach Educator, Outreach Worker, Peer Educator, Promotora, Promotoras de salud, Sevika, Shastho karmis, Shastho shebika, Shasthya Shebika, Socorrista, Village Drug‐kit Manager, Village Health Helper, Village Health Volunteer, Village Health Worker, Volunteer Health Worker, Voluntary Health Worker
Primary Health Care, Comprehensive Primary Health Care, Selective Primary Health Care, Vertical Programme, Primary Care
Community Perception, Community Perspective, Attitude to Health, Opinion of Community, Community Feelings, Community Experience, Community Satisfaction, Community acceptance, Uptake, Service Utilisation, Barrier to Health, Quality of Health care
Rural area, Remote Area, Rural Population, Rural Community, Rural Catchment, Rural Health Service, Rural Health, Rural Health Centre

This search strategy was initially formulated for PubMed. It was later adapted for use in other databases (Supporting Information S1). We conducted a comprehensive search of peer‐reviewed original research articles published in English from January 1990 to June 2024, utilising seven distinct electronic academic databases, including EBSCOhost CINAHL Ultimate, Cochrane Library, ProQuest Central, Scopus, Ovid MEDLINE, APA PsycINFO on Ovid and PubMed to ensure a thorough and systematic search. The selection of databases was based on accessibility of pertinent resources from the University of Waikato's Library. Eligibility criteria for inclusion and exclusion of articles were developed using the Population, Interest, Comparison and Outcome (PICO) framework outlined in Table 2.

**TABLE 2:** Eligibility criteria.

PICO criteria	Inclusion	Exclusion
Population	Community members of rural areas	The study population belongs to urban areas
Interest	The community members' perception of the Primary Health Care services delivered by Community Health Workers and the factors influencing that perception	The Perception of Community Health Workers was recorded instead of the community members' perception
Comparison	No comparison	
Outcome	Improved or decreased satisfaction (acceptance, uptake and utilisation of services) of community members for Primary Health Care services delivered by Community Health Workers in rural areas	

### 2.3 Screening Process

**FIGURE 1:**
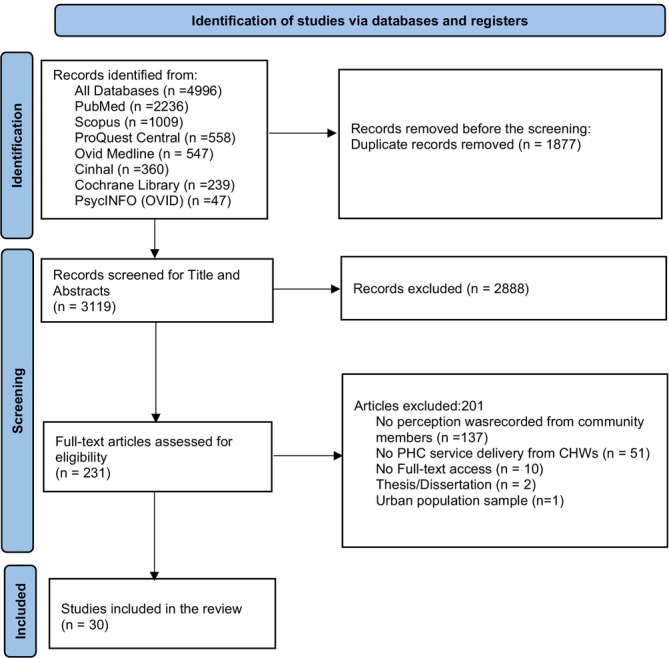
PRISMA flow diagram [49] showing the study selection process.

A comprehensive search identified 5471 articles (Figure 1). After applying the English language and time duration filter, we narrowed it to 4996 articles. Database searches were performed by one reviewer (NA). After database searches, results were exported in a format compatible with EndNote. Following de‐duplication in EndNote, the articles were uploaded to Rayyan (a systematic review platform) [50], where further automatic and manual de‐duplication was done. Using Rayyan, two independent reviewers (N.A. and M.D.) screened eligible articles using eligibility criteria (Table 2), first evaluating titles and abstracts and then conducting the full‐text screening (Figure 1). Disagreements were discussed between the two, and if unresolved, a third reviewer (Author H.C. or R.S.) made the final decision.

### 2.4 Quality Appraisal

The quality of all eligible articles after the full‐text screening was appraised (Supporting Information S2) using the Mixed Methods Appraisal Tool (MMAT) 2018 [51]. The MMAT 2018 was selected to encompass the broad spectrum of study designs considered in the review and due to its proven content validity [52] and sufficient reliability [53]. Two reviewers (N.A. and M.D.) appraised the studies as per the instructions by the authors of MMAT 2018 [54], and any disagreement was resolved through discussions.

### 2.5 Data Extraction and Synthesis

Two independent reviewers (N.A. and M.D.) extracted the data from eligible articles using a standard procedure. This systematic review used narrative and descriptive data synthesis methods to analyse and present our findings [46]. We systematically organised and extracted study characteristics and findings from each included study. A tabular format was used to display the characteristics of all included studies (Table 3). To compare and categorise the factors influencing community perceptions of CHWs delivering PHC services in rural settings, we enlisted them in Table 4. For the descriptive synthesis, we created another table (Table 5) highlighting the frequency of these factors across various studies. In addition, we employed narrative synthesis to analyse the findings, uncovering contextual patterns, thematic clusters and insights that simple frequency counts might miss. We further organised the factors into thematic clusters to demonstrate their interrelations. To enhance clarity, we have adopted the use of “CHWs” as a standard term to collectively refer to the various designations of community health workers across different nations, as indicated in the relevant literature.

We followed the National Institute for Health and Care Excellence (NICE) definition of community: “A community is a group of people who have common characteristics or interests. Communities can be defined by geographical location, race, ethnicity, age, occupation, a shared interest or affinity (such as religion and faith) or other common bonds, such as health need or disadvantage. People who are socially isolated are also considered to be a community group” [85]. Based on the WHO's definition of community health workers (CHWs) and the concept of community taken from the above definition, we consider all recipients of services provided by CHWs to be community members, and the area where CHWs deliver services as the catchment area of the CHW. Therefore, we will use the term “community members” as recipients of services delivered by CHWs throughout this systematic review.

There is no universally accepted definition of rural areas [44, 86]. In this systematic review, we have categorised a region as rural based on the terminology used in the articles; if the articles referred to the research area or population as rural, we classified it accordingly. If the term “rural” was not explicitly mentioned, we will rely on the categorisation used by the host countries to determine whether the research was conducted in a rural area.

### 2.6 Ethics Statement

This is a systematic review, and the reviewers used publicly accessible documents as evidence. There was no requirement for an institutional ethics approval before commencing a systematic review.

## 3 Results

### 3.1 Study Characteristics

This systematic review includes thirty studies. Characteristics of included studies are summarised in Table 3. These studies used a variety of methodologies (Qualitative design *n* = 18, Quantitative design *n* = 7, Both qualitative and quantitative methodologies *n* = 3, and Mixed‐methods study *n* = 2). Among the included studies, 14 were on generalist CHWs. We were unable to determine the type of CHWs in the remaining studies using the information provided in the articles.

**TABLE 3:** Summary of characteristics of peer‐reviewed published studies [55, 56, 57, 58, 59, 60, 61, 62, 63, 64, 65, 66, 67, 68, 69, 70, 71, 72, 73, 74, 75, 76, 77, 78, 79, 80, 81, 82, 83, 84] included in the systematic review.

S.#	Country	Author and year	Name and type of CHW^a^	Objectives of study/research questions	Service delivery by CHWs	Study design, sample population, sample size and sampling technique	Study limitations^b^
1	Bangladesh	Puett et al. (2013) [55]	Community Health Workers (CHWs) Type: Generalist	“To measure CHWs' technical competence in managing cases of severe acute malnutrition (SAM) according to a treatment algorithm.” “To examine the subjective aspects of quality of care by assessing elements of CHW service delivery that was valued by caretakers.”	Community‐based management of acute malnutrition	Mixed‐Methods Study The qualitative part: Focus Group Discussions (FGDs) with caregivers, *n* = 29. The quantitative part included a Cross‐sectional Survey of CHWs and case management observations, *n* = 55	Only the qualitative part of this study matched the requirements of this systematic review. We extracted findings from FGDs with caregivers. Data were gathered in the dry season when the prevalence of SAM was low. An increase in cases during the rainy season could potentially affect the quality of care
2	Brazil	Atkinson and Haran (2005) [56]	Community Health Workers (CHWs) Type not determined from this article	“To explore possible determinants of user satisfaction based on a broad conception of the health care system involving both individual and district scale variables and to add to the limited knowledge and empirical study about such determinants.”	Family Health Programme	Quantitative Study Survey (*n* = 4600 women; the analysis was restricted to those women who reported use of a health facility in the past 3 months, *n* = 2956) Purposive sampling	The study focused on women who had accessed health services in the past three months. Those who did not, possibly due to dissatisfaction or barriers, were excluded, which may bias the results
3	Eswatini	Walker et al. (2020) [57]	Rural Health Motivators (RHMs) Type: Generalist	“To understand the role of RHMs in decentralised HIV (Human Immunodeficiency Virus)/TB (Tuberculosis) activities.” “To understand the role of RHMs by key stakeholders, including community members, local leadership and government structures.” “To know RHM's experience of programme recognition.”	HIV/TB activities	Qualitative Study with Exploratory Approach 19 In‐depth Interviews (8 With RHM, 2 with Ministry of Health (MoH) Nurse, 2 with Community Leader, 2 with CHW Trainer, 2 with Medecins Sans Frontieres (MSF) Staff, 2 with MoH (Key Informant), 1 with Ministry of Tinkhundla Administration and Development (MTAD) Key Informant). 4 FGDs (*n* = 29); 2 FGDs with women, 1 with men, 1 with people living with HIV (Women). 1 Group discussion with RHMs, *n* = 5 (Using participatory methods) 1 Group Discussion with men, *n* = 3 4 Observations of RHM Purposive Sampling	From this study, we extracted findings from the FGD with men and women and interviews with community leaders. The rest of the data was not relevant to our focus in this review. According to this study, communities frequently misinterpret the responsibilities of RHMs, anticipating them to carry out duties that exceed their official role, such as handing out food or offering material support (for example, first aid supplies). We do not include this aspect in our findings
4	Ethiopia	Adamu (2012) [58]	Health Extension Workers (HEW) Type: Generalist	“To explore factors that contributed to the participation of rural communities in health education.”	Health Education	Qualitative Study 3 FGDs (8–10 participants in each FGD). 6 Interviews: with HEWs Purposive and Snowball sampling technique: Community members Convenience Sampling: HEWs	This study collected data from HEWs and community members and we extracted information from community members only. The research utilised purposive and snowball sampling methods to select participants, which may not result in a fully representative sample of the rural population
5		Agraw et al. (2007) [59]	Community‐based reproductive health agents (CBRHAs) Type not determined from this article	“To assess sustainability and determine the factors that cause variations in the success of CBRHP.”	Reproductive Health (RH) Services	Comparative cross‐sectional study using both quantitative and qualitative methods for data collection. Quantitative Part: Cross‐sectional survey (Women of 15–49 years, *n* = 792)‐Simple random sampling Qualitative part: Key informants' interviews with programme coordinators and FGDs with CBRHAs (number of participants not determined)‐ Multistage sampling technique	The quantitative part of this study is related to the aims of this review, so data only from this part was extracted
6		Medhanyie et al. (2012) [60]	Health Extension Workers (HEWs) Type not determined from this article	“To study the extent to which specially trained community health workers have contributed to the improvement of utilisation of maternal health services by rural women in Ethiopia.” “To assess the utilisation of maternal health services by women in rural villages in Ethiopia.”	Maternal Health Services	Quantitative Study Cross‐sectional survey (*n* = 725 households‐women with under five children)	In this study, information of our interest was only in one section: association with respondents' characteristics with the utilisation of maternal services
7		Shaw et al. (2017) [61]	Health Extension Workers (HEWs) Type not determined from this article	“To explain the integrated community case management (ICCM) delivered by HEWs through examining the care‐seeking practices and treatment for sick children in two rural districts in the Oromia Region of Ethiopia.”	Integrated community case management (iCCM)	Qualitative Study 78 Interviews (40 with mothers, 16 with fathers, 10 with HEWs and 12 with volunteers). 16 FGDs, *n* = 132 mothers (6–12 participants in each FGD). Purposive sampling	Information relevant to our systematic reviews was only in the perception and choice of care provider part
8		Shaw et al. (2015) [62]	Health Extension Workers (HEWs) Type: Generalist	“Patterns of care seeking from HEWs delivering evidence‐based treatments for sick children in community‐based settings and their social and economic determinants in the context of scale‐up of Integrated Community Case Management (iCCM).”	Integrated Community Case Management (iCCM) vs. Routine Community Case Management (CCM)	Quantitative Study Cross‐Sectional Survey (*n* = 2248; Caregiver of an under‐five child sick with diarrhoea, fever and/or suspected pneumonia in the last 2 weeks) Stratified two‐stage cluster sampling	This study attempted to control for confounding factors; however, intricate factors such as socioeconomic status, educational attainment and geographic access might still influence the findings and result in residual confounding
9		Tesfaye et al. (2014) [63]	Health Extension Workers (HEWs) Type: Generalist	“To examine how the promotion of community maternal and newborn health (CMNH) family meetings and labour and birth notification contributed to increased postnatal care within 48 h by skilled providers or health extension workers.”	Training in Maternal and Newborn care through Community Maternal and Newborn Health (CMNH) family meetings	Quantitative Study Cross‐Sectional Surveys Baseline survey, *n* = 1027 women Endline Survey, *n* = 1019 women	The study might have been affected by recall bias and inadequate confounding factor control. The regression model adjusted for women's use of birth care providers, limiting the impact of CMNH meeting attendance
10		Yirgu et al. (2020) [64]	Health Extension Workers (HEWs) Type not determined from this article	“To explore the role of health care providers in women's family planning decision‐making in Ethiopia.”	Family Planning Services	Qualitative Study 30 in‐depth interviews (*n* = 120, 12 couples and 6 single women in each of 4 sites) 10 FGDs (*n* = 80, 10 participants (women aged 15–49 and men aged 18+) in each FGD) with semi‐structured interview guides. Purposive Sampling	The use of community‐based convenience sampling might have enabled women with more intense experiences related to family planning to choose to participate in the study; nevertheless, since this research primarily focused on women's experiences regarding pregnancy, family planning and sexuality, this is different from
11	Ghana	Baiden et al. (2007) [65]	Lay Counsellor Type not determined from this article	“To assess approval for the use of lay counsellors to promote community‐based voluntary counselling and testing for HIV and the extent of HIV/AIDS‐related stigma in the Kassena‐Nankana district of rural northern Ghana.”	Community‐based voluntary counselling and testing for HIV (CBVCT)	Both quantitative and qualitative methodologies. Quantitative: Cross‐sectional survey, *n* = 403 Qualitative: 13 FGDs *n* = not determined (7–10 participants in each FGD), 5 FGDs with male and 4 FGDs with female adolescents (15–24 years) and 2 FGD each with male and female adults (25–65 years). Random sampling	The response may be influenced by prior knowledge of what the respondent believes would be an acceptable answer
12		Stephens et al. (2020) [66]	Community Health Workers (CHW) Type not determined from this article	“To explore the perceived role and value of CHWs in supporting family planning services and addressing associated barriers to family planning use.”	Family Planning services	Qualitative study: in‐depth interviews with women *n* = 33 (purposive sampling) and with active CHWs *n* = 30 (convenience sampling)	We extracted data only from interviews with women, which aligns with the aim of this review. Interview guides were not translated into Twi to allow for unscripted interviews by research assistants. The use of English‐speaking researchers may have limited the ability to capture nuances and led to socially desirable responses. Positive responses from CHWs could be influenced by the presence of North American student researchers. Potential biases in the analysis were introduced by the race, nationality and privilege of the student researchers, despite their training in ethics and collaboration with Ghanaian colleagues
13	India	Akilan et al. (2014) [67]	Village Health Workers (VHW) Type not determined from this article	“To review the project (of early detection of hearing loss among infants and young children) by examining the caregiver perception from the mothers of children who have undergone hearing screening regarding the service provided.”	Hearing screening using Oto Acoustic Emissions (OAE) equipment	Qualitative Study. 9 FGDs *n* = 83 (Mothers of infants and children) Random sampling	The involvement of VHW in contacting mothers would have influenced responses
14		Blanchard et al. (2024) [68]	Accredited Social Health Activists (ASHAs) Type: Generalist	“To explore how people's understandings of higher and lower ‘socio‐economic position’ were informed by social relations in the spaces where they live, interact and access resources, and the ways in which these socio‐spatial processes influenced equity in coverage of community‐based maternal and newborn health programmes there.”	Community‐Based Maternal and Newborn Health Programme	Qualitative Study (a part of mixed‐methods doctoral study), 12 FGDs, *n* = 134, 8 FGDs with women (*n* = 88) and 4 with ASHAs (*n* = 46), Purposive Sampling	From this study, only information from the FGDs with women was relevant to this review. The study struggled to recruit participants from small villages and varied socio‐economic backgrounds despite diverse engagement. It used conversation‐based methods, which might capture reported actions better than actual behaviours due to biases
15	Kenya	Juma et al. (2015) [69]	Community Health Workers Type: Generalist	“To describe the perception of women towards family planning service provision by CHWs in four rural districts of western Kenya.”	Family Planning	Quantitative Study, Cross‐Sectional Baseline Survey, *n* = 963 (Women 15–49 years)	The analysis was restricted to 963 valid responses from the 1997 women surveyed, which could diminish the sample's representativeness and restrict the applicability of the results to all women in the research area
16		Kamau (2020) [70]	Community Health Volunteers (CHVs) Type: Generalists	“To describe the experiences of CHVs of participating in a community‐based approach for Iron and Folic Acid Supplements (IFAS).” “The specific objectives of the study were to (1) describe perceived benefits. (2) identify challenges experienced and (3) describe recommendations for CHVs, nurses and pregnant women of participating in a community‐based approach for IFAS.”	IFAS tablets distribution and education to pregnant women	Qualitative Study, 19 In‐depth interviews (8 Pregnant Women (random sampling), 2 Nurses, 9 CHVs)	Our focus in this study was on data extraction from interviews with pregnant women only
17		Kibel et al. (2022) [71]	Community Health Volunteers (CHVs) Type: Generalists	“To determine whether CHV‐delivered point‐of‐care Urine pregnancy test (UPT), post‐test counselling, and referral to care is an acceptable and feasible strategy to increase the uptake of ANC, FP and other reproductive healthcare services, both to CHVs delivering the intervention and to women and men in the community.”	Urine pregnancy test (UPT), post‐test counselling and referrals to Antenatal care (ANC), Family planning (FP) and other reproductive healthcare services	Qualitative Study Pre‐intervention: 12 FGDs (4 with CHVs, 4 with women and 4 with men) 7–9 participants per FGD, Post‐intervention: 4 FGDs (4 with CHVs) 7–9 participants per FGD, Convenience Sampling	Data relevant to our focus in this review was only on pre‐intervention FGDs with women and men. Post‐intervention data were not relevant to our aim for this systematic review
18		Rogers et al. (2022) [72]	Community Health Workers (CHWs) Type: Generalists	“To explore how socioeconomic characteristics, household (HH) behaviours and access to community health services predict under‐5 care utilisation for fever, diarrhoea and Acute Respiratory Infection (ARI) symptoms in rural Migori County.”	Integrated Community Health Intervention	Quantitative Study (Cross‐Sectional Study), *n* = 4137 (female or male head of household)	In this study, our data extraction was confined to the predictors of care utilisation only
19	Malawi	Ndambo et al. (2024) [73]	Community Health Workers (CHWs) Type not determined from this article	“To explore perspectives of community and facility stakeholders on the enablers and challenges of the CHW role in community‐based PHC in Neno Districts.”	Health education, screening, linkages, patient follow‐up and psychological support	Exploratory Qualitative Study, 8 FGD, *n* = 92 (11–12 participants in each FGD), 45 community members (purposive sampling) and 47 Primary health care workers, Convenience sampling	Our review focus is on community members, so we only extracted data from community members' FGDs
20	Myanmar	Watt et al. (2016) [74]	Community Health Workers (CHWs) Type: Generalists	“To explore factors affecting Village Health Workers (VHW), i.e., Auxiliary midwives (AMW), Community Health Workers (CHW) service usage in Myanmar, by focusing on perspectives and reported experiences of service users, community providers and national policymakers related to volunteer health workers in a rural area of Myanmar.” Research Question: “How do service users experience accessing and receiving community‐based healthcare?”	Health Education Sanitation and Immunisation, Treatment of minor injuries and illnesses Coordination with health facilities for early referral	Qualitative Study, In‐depth semi‐structured interviews, *n* = 105 (54 Service users, 9 CHWs, 6 Auxiliary Midwives (AMW), 4 Midwives and 8 Village Health Volunteers, 14 staff from 2 UN agencies, 7 from International NGOs and 1 from National NGO)	Out of 105 interviews, data relevant to this review was from 54 interviews with service users
21	New Zealand	Ratima et al. (1999) [75]	Hauora Runanga Māori Community Health Workers Type not determined from this article	“To assess whether the long‐term benefits of the programme extend beyond reduced asthma morbidity and the extent to which any additional benefits may be related to the partnership approach employed by the programme.”	Support along with doctors at Māori community meetings at Marae for asthmatic patients	Qualitative Study, Standardised open‐ended Interviews, *n* = 25 (after dropouts)	Only 68% of enrolled participants actually took part in the study. Out of the 47 who participated, three had passed away and 19 had moved out of the area and could not be contacted; they did not attend at least two interviews
22	Nigeria	Okereke et al. (2020) [76]	Community Health Extension Workers (CHEWs), Junior Community Health Extension Workers (JCHEWs) and Community Health Officers (CHO) Type not determined from this article	“To investigate female clients' gender preferences for frontline health workers who provide maternal, newborn and child health (MNCH) services at the primary health care (PHC) level using Bauchi and Cross River states.”	Maternal, newborn and child health services (MNCH)	Quantitative Study (Cross‐sectional study) Purposive sampling, *n* = 256 (women)	In this study, front‐line workers also included workers other than CHWs. However, we only identified findings as per the aims of our systematic review
23	South Africa	Grant et al. (2017) [77]	Community Health Workers (CHWs) Type: Generalist	“To explore CHW acceptability and to identify possible barriers to successful implementation of this strategy.”	Maternal, child and women's health (MCH) services	Qualitative Study, 19 Focus group discussions *n* = 143 (3 FGDs with mothers of under 5 years children *n* = 25, 3 with men with under 5 years children in household *n* = 21, 3 with grandmothers of under 5 years children *n* = 19, 5 with CHWs *n* = 41, 5 with professional nurses *n* = 37) Purposive sampling	We extracted findings from data resulting only from FGDs of mothers, grandmothers and men, as well as interviews with women
24		Wilford et al. (2018) [78]	Community Health Workers (CHWs) Type: Generalist	“To explore the quality of Maternal and Child Health (MCH) services and health promotion messages provided to women and infants by CHWs visiting households.”	Maternal and child health (MCH)	Qualitative Study, Observations of household visits by CHW (30 household visits) In‐depth Interviews *n* = 45 (30 women and 15 CHWs) Convenience sampling	Information from observation of household visits by CHWs and interviews with CHWs was not relevant to our review. We only extracted findings from interviews with women
25	Tanzania	Rafiq et al. (2019) [79]	Community Health Agents (Wawezeshaji was Afya ya Jamii‐WAJA) Type not determined from this article	“To understand the community's reception of community health workers in Connect intervention areas using an ethnographic approach.”	The integrated and comprehensive package of services (Reproductive, Maternal, Newborn, Child and Adolescent Health‐RMNCAH, disease surveillance, preventive and curative services and connecting households to health facilities)	Qualitative Study 88 In‐depth interviews and 24 FGDs (4 with women with child under 1, 4 with men with children under 5, 6 with WAJA, 4 with members of village government, 4 with WAJA village supervisors, 2 with WAJA clinical supervisors) Ethnographic: Observation and participation (*n* = 6 CHWs)	Our findings were extracted from data of 4 FGDs with women with a child under 1 year and 4 FGDs with men with a child under 5 years. The rest of this study was beyond the scope of our review
26		York et al. (2014) [80]	Community Drug Distributors (CDDs) and Community Health Workers (CHWs) Type: Generalists	“To explore community‐perceived factors related to participation in and sustainability of the CDTI program in southwest Tanzania.”	Community‐directed treatment with Ivermectin (CDTI)	Quantitative and Qualitative Study, Quantitative part: Cross‐Sectional Survey (*n* = 456) Qualitative part: Seven FGDs (*n* = 42), including 3 FGD (*n* = 15) with women who took the medication, men who took the medication and men who did not take the medication and 4 FGDs (*n* = 27) with women who took the medication, women who did not take the medication, men who took the medication and men who did not take the medication Eight Semi‐structured Interviews (5 with community‐directed distributors and 3 with community health workers)	In this study, interview data was not related to the aims of our review. A research assistant misinterpreted a survey question, leading to the removal of 24 surveys
27	Uganda	Batte et al. (2021) [81]	Community Health Workers (CHWs) Type: Generalists	“To understand the acceptability of the implementation of a patient‐centred education intervention delivered by CHWs using the PocketDocket among (people living with HIV/AIDS (PLWHA)) with hypertension in Nakaseke, a rural Uganda setting.”	Provision of education to patients using the hypertension module of PocketDocket (a validated patient booklet)	Qualitative Study, In‐depth interviews (IDIs) *n* = 22 (14 with people living with HIV/AIDs and Hypertension and 8 with CHWs) Three FGDs: 2 with people living with HIV/AIDS and Hypertension and 1 with CHWs. Purposive sampling	In this study, participants of FGDs and interviews are both people living with HIV/AIDs and hypertension and CHWs. However, we only focused on data from people living with HIV/AIDs and hypertension to identify our findings. The study had more female participants, possibly influencing perceptions. Positive responses about CHWs from the same community might introduce bias
28		Buchner et al. (2014) [82]	Community Health Workers (CHWs) Type not determined from this article	“To assess stakeholder perceptions of the Health Child Uganda (HCU) Integrated Community Case Management (iCCM).”	Integrated community case management (iCCM)	Qualitative study, 15 Semi‐structured FGDs *n* = 101 participants (6 with caregivers *n* = 35, 6 with CHWs) *n* = 47, 1 with Health Centre Staff *n* = 7 and 2 with Local Leaders *n* = 12, 5 Semi‐structured Key informant interviews (1 with the sub‐county chairman, 1 with the sub‐county chief, 1 with the district health officer, 1 with Health and Social Services and 1 with the District Council Leader)	We extracted findings from information resulting from 6 FGDs with caregivers, *n* = 35. The rest of the data in this study was beyond the scope of our Systematic Review. Only those community members who sought services from CHWs were included in the study
29		Waiswa et al. (2008) [83]	Community Volunteer Type not determined from this article	“To explore the acceptability and barriers to the recommended evidence‐based practices and to home‐visiting by a community volunteer to improve practices along the continuum of maternal and newborn care for better neonatal outcomes.”	Maternal and child health care	Qualitative Study, 10 FGDs (6 FGDs with mothers or grandparents, 2 with fathers and 2 with childminders. Number of participants not reported) 10 Informant Interviews (6 with Health Workers and 4 with Traditional Birth Attendants)	The interviews section of this qualitative study was not in the scope of our review. Secondly, the study focused on care practices for newborns among women who had recently given birth. Maternal issues were only addressed if they impacted the newborn
30	Zambia	Henning et al. (2020) [84]	Community Health Assistants (CHAs) Type not determined from this article	“To explore the lived experiences of adolescent mothers and the services provided to them by community health assistants (CHAs).”	Community Education, diagnosis of common diseases, Provision of restricted medical treatment	Mixed‐Methods design, 8 FGDs (*n* = 60 mothers), 34 Key informants Interviews: 12 with adolescent mothers and 22 with CHAs, Surveys: *n* = 40 CHAs, Purposive Sampling	In this study, we identified findings only from data resulting from FGDs with mothers and 12 interviews with mothers. Although it is a mixed‐method study, the type of mixed‐methods study is not specified

^a^ Community Health Worker.

^b^ Limitations refer to aspects identified in these studies that do not align with the objectives of this systematic review. While certain studies contained components outside the scope of this review, there were also sections within the same studies that were relevant; data extraction was conducted exclusively from those pertinent parts.

### 3.2 Study Quality

Mixed Methods Appraisal Tool (MMAT) 2018 was used for appraisal. Included studies met the required criteria of inclusion (Supporting Information S2).

### 3.3 Geographic and Economic Distribution of Included Studies

In this review, the included studies are from 15 different countries spread across four continents. Twenty‐five studies are from Africa, three from Asia, and one each from South America and Oceania. Ten countries are from Africa, three from Asia, and one from each South America and Oceania. Among these countries, only one is a developed nation [87]. Given resource differences, factors like accessibility—such as travel distance, transportation infrastructure and financial barriers—have greater implications in less developed settings, where economic constraints often limit physical access to CHWs and facilities. Barriers to health care are different in developing nations and developed nations. Therefore, when considering the findings of this review, the particular context should be considered.

### 3.4 Factors Affecting Perception of Community Members

Identified Factors affecting the perception of community members regarding the service delivery of primary health care (PHC) services by community health workers (CHW) in rural areas are listed in Table 4. Table 5 shows the frequency of studies supporting the most frequent factors influencing the perception of community members.

**TABLE 4:** Factors affecting community perception identified from peer‐reviewed research studies [55, 56, 57, 58, 59, 60, 61, 62, 63, 64, 65, 66, 67, 68, 69, 70, 71, 72, 73, 74, 75, 76, 77, 78, 79, 80, 81, 82, 83, 84].

S.#	Country	Author and year	Factors affecting community perception	
			Positive effect on perception	Negative effect on perception
1	Bangladesh	Puett et al. (2013) [55]	Community Health Workers (CHWs)^a^ from the same community as caregivers. Interpersonal skills and technical competence of CHWs. CHWs who share knowledge with caregivers. Practical demonstrations from CHWs. Regular house visits by CHW and follow‐up mechanisms. Provision of ready‐to‐use therapeutic food (RUTF) for children by CHWs. Provision of advice for children to caretakers. Treating caregivers nicely. Checking children for problems (Temperature, oedema, breathing count)	
2	Brazil	Atkinson and Haran (2005) [56]	Familiarity with CHW. Household visit by CHW. Good healthcare outcome. Had a health problem to be seen	
3	Eswatini	Walker et al. (2020) [57]	Gender of CHW (Female CHWs s for women and male CHWs for men)	
4	Ethiopia	Adamu (2012) [58]	Discussion about health education objectives during house‐to‐house visits. CHWs being members of the same community. The female gender of CHWs. Peer influence (friends and models, families). Experiencing changes in friends' and neighbours' houses and lives after participating in the health education programme. Services at places convenient to community members (houses or fields where community members work). Attending health education lessons does not affect daily routine work	
5	Ethiopia	Agraw et al. (2007) [59]	Participation of community members in the selection of CHWs. Acceptance of the CHWs. Awareness of the presence of CHWs. Discussion with CHWs. Satisfaction with CHWs. Awareness of the presence of village health committees (that monitor the activities of CHWs). Ever talked to CHWS regarding reproductive health issues	
6	Ethiopia	Medhanyie et al. (2012) [60]	Literate community member. Listening to a radio. Good participation in Income Generating Activities (IGAs). Working towards graduation or graduating as a model family	
7	Ethiopia	Shaw et al. (2017) [61]	Explanation of culturally and socially understood meanings of a child's illness by CHW. Lay referral networks. Previous child illness experiences. The cultural taboo of not leaving home by caregivers of very young children	Unavailability of CHWs, particularly at night and on weekends. Difficult accessibility (e.g., long distances, geographical barriers and challenges transporting unwell children). The preference of influential family members (especially the elderly) and community members. Distrust in government‐run health programmes due to ethnic conflicts in the country
8	Ethiopia	Shaw et al. (2015) [62]	Previous use of services for child illness. Awareness of the availability of treatments for Child illness. The child with reported pneumonia	Perception of the seriousness of Illness (Perception that the child's illness is not severe). Negative perception regarding the availability of services (The perception that the service delivery site is not open). Non‐availability of drugs with CHWs. Too far to access (difficult accessibility). Poor service from CHWs. Preference for informal treatment. No transportation. Mothers with some formal education. Children under 1 year of age. Type of illness (Child reported with diarrhoea only)
9	Ethiopia	Tesfaye et al. (2014) [63]	*(Perception regarding Postnatal Care services from CHWs)* Community members' residence in the Amhara region of Ethiopia. Receiving antenatal care from CHWs. Attending two or more community maternal and newborn health (CMNH) family meetings. CHWs' mobile phone Number on a household, friend's, or neighbour's phone. The most recent birth attended by a CHW	
10	Ethiopia	Yirgu et al. (2020) [64]	Trustable CHWs. Credible CHWs. Knowledgeable CHWs. Detailed information regarding service delivery from CHWs	Fear of the family planning procedure. Distress and discomfort in interaction with CHWs. Manipulation by the CHWs to choose long‐acting methods like implants. Not receiving the preferred family planning method. Resistance from the partner
11	Ghana	Baiden et al. (2007) [65]	Availability of HIV rapid kits. Availability of service at home, any convenient place within the community or other locations outside of the health facility. Accessibility. Socio‐cultural familiarity. Native lay counsellor. Same sex CHW. Same age or older CHW	Stigma, fear and Misconceptions about HIV. Breaches in confidentiality
12	Ghana	Stephens et al. (2020) [66]	Discussion about family planning with CHWs. Easy accessibility to CHW. Familiarity of household members with CHWs. The feeling of comfort with CHWs. Confidentiality. Provision of services at home. Frequent contact with CHW. Trust in CHW	Stigma regarding family planning
13	India	Akilan et al. (2014) [67]	Receiving prior information regarding intervention from preschool teachers. Provision of details of service. Knowledge of other children used this intervention. Familiarity with CHW. Service provision at home. Free service. Community involvement	
14	India	Blanchard et al. (2024) [68]	Women from lower socioeconomic positions (SEP)	Difficult accessibility (Long distance)
15	Kenya	Juma et al. (2015) [69]	Reading a newspaper or a magazine. Listening Radio. Watching Television. Level of knowledge about family planning benefits for the child and mother. Young women (15–24 years old). Middle‐aged women (25–39). Having 2–5 Children. Ethnicity (Luo). Low education (less than secondary level). Religion	Religion. Not discussing FP with the spouse. Less than two children or six or more children. Ethnicity (Luhya, Teso). Old age (40–49) of women
16	Kenya	Kamau (2020) [70]	Home visits by CHW. The regular supply of Iron and Folic Acid Supplements (IFAs) at home. Health education provision at home. A follow‐up visit allows for discussing any other health problems	Delayed home visits by CHW
17	Kenya	Kibel et al. (2022) [71]	Home visit by CHW. Free services. Easy accessibility (nearby location of CHW). Younger and older women (who experience stigma in pregnancy). Women have limited freedom to leave the house. Limited health education. Women who wish to obtain a Urine pregnancy test (UPT) without informing their spouse or other family members. Detecting pregnancy early helps start prenatal care early and receiving negative pregnancy test results helps start family planning on time. Opportunities to connect clients with other reproductive health services, such as infertility treatment, HIV care and mothers' groups (Chamas), and to enrol in the Kenya National Health Insurance Fund. Trust in CHW. Respect for CHW. Confidentiality	A breach of confidentiality. Biases and conflicts between community members and CHW. The perception that UPT kits could be used for HIV testing. Intervention (Family Planning) is unacceptable to the community, traditions, or religion. Perception of side effects of family planning. Opposition from spouse
18	Kenya	Rogers et al. (2022) [72]	Male partner attendance at Antenatal clinic (ANC) visit. The head of the Household having secondary education. Household visit by CHW. Households previously experienced the death of a child under five years old	History of health facility delivery. Large household size
19	Malawi	Ndambo et al. (2024) [73]	Household visits (provision of health education) by CHW. Trustworthy CHW. Supportive care beyond assigned duties (providing money for or physically carrying a patient to a referred health facility). Social interactions of CHWs	Lack of capacity of CHW. Religious and traditional beliefs
20	Myanmar	Watt et al. (2016) [74]	Experience and skills of CHWs. Personal relationships with CHWs. Perceived effectiveness of drugs provided by CHWs. Services availability in the neighbourhood. Free services. Flexibility in accessing CHW. Availability all the time. Referral service from CHWs. Trust between CHWs and service users. Familiarity with CHWs. Own or others' previous service users' experience with CHW	Non‐availability of drugs with CHWs. Insufficient quality. Complex health issues (chronic conditions, childbirth)
21	New Zealand	Ratima et al. (1999) [75]	Provision of a relaxed atmosphere at facilities. Provision of information in a way that is easy to understand. Marae‐based clinic managed by Māori (perceived as friendly). Māori programme workers (perceiving them as their own people). Free services	Religion, distance and house boundness
22	Nigeria	Okereke et al. (2020) [76]	Female gender of CHW	
23	South Africa	Grant et al. (2017) [77]	Trust in CHWs. Confidentiality. Client permission for household visits by CHW. Female gender of CHW. CHW from an area other than that of the client (Some participants perceive them as more trustworthy). CHWs from the same community as the client (other participants perceive them as more trustworthy). Competencies of CHWs as a service provider. Familiarity with CHWs	Lack of confidentiality and trust. Male gender of CHW
24	South Africa	Wilford et al. (2018) [78]	Referral to a clinic. Household visits. Accessibility. Confidentiality. Provision of information in a way which is easy to understand. CHW living in a nearby location. Being from the same community. Sufficient time for discussion during household visits. Support for HIV patients and referral	
25	Tanzania	Rafiq et al. (2019) [79]	Availability of medicine. Curative services. Female gender of CHW. Male gender of CHW (for younger women). Social relationship with CHW	Unavailability of medicine with CHW. Lack of curative services
26	Tanzania	York et al. (2014) [80]		Lack of knowledge and information about the drug used in service delivery. Fear of taking drugs. The perception that individuals are not sick and, therefore, do not require any medication. Disagreement regarding the method for calculating the dosage of ivermectin. Lack of trust due to CHWs are not medically trained
27	Uganda	Batte et al. (2021) [81]	Use of easy‐to‐understand, non‐technical terminologies. Affinity with CHWs due to a similar lifestyle. Rapport of CHW for the delivery of other health services Perception of a good outcome of service delivery. Material in the local language. Considering health as a priority	Inaccessibility
28	Uganda	Buchner et al. (2014) [82]	Easy Accessibility round the clock. Availability at all times. Reliability. Trustworthy CHW. Compassionate care. Good response from the health centre with CHW's referral. Improved health outcomes. Less time needed for service utilisation. Less cost needed for service utilisation	Limitation of treatment to under‐five children only. Uneducated CHW. Misdiagnosis by CHW
29	Uganda	Waiswa et al. (2008) [83]	Community Volunteer be: Of a Female Gender. Permanent resident in the area. Literate. Well‐behaved and respected in the community. Able to walk long distances. Good‐tempered. Knowledgeable. Punctual. A good listener. Confident. Experienced in parenting. Married. Kind‐hearted person	
30	Zambia	Henning et al. (2020) [84]	Female gender of CHW. Stigma. Confidentiality	

^a^ To enhance clarity, we have utilised “CHWs” as a standard term to refer to the various names of community health workers across different countries, as presented in the relevant articles.

**TABLE 5:** Most frequently identified factors affecting the perception of community members.

S.#	Factors influencing the perception of community members	Number of studies reported this factor
1	Accessibility to CHW^a^	10
2	Trust in CHW	10
3	Availability of services convenient to community members	8
4	Gender of CHW	8
5	CHW from the same community	8
6	Household visit by CHW	7
7	Competency of CHW	7
8	Confidentiality	7
9	Familiarity with CHW	6
10	Previous experience with CHWs	5
11	Influence of partner/family	5
12	Perception of quality of healthcare	5
13	Availability of medicine/drugs	4
14	Availability of free services	4
15	Stigma regarding service delivery	3
16	Availability of referral services from CHWs	3

^a^ Community Health Worker.

### 3.5 Thematic Clusters Influencing Community Perceptions of CHW‐Delivered PHC Services

We identified three interrelated thematic clusters that influence community members' perception of CHW service delivery: (1) the structural and service delivery group, (2) the trust group and (3) the identity and sociocultural group. These three clusters illustrate overlapping areas. They are not distinct categories. It indicates that various factors collectively shape perceptions of community members regarding CHWs' service delivery. Table 6 illustrates the classification of factors within the Thematic Cluster.

**TABLE 6:** Hierarchy of influence on perceptions.

Cluster	Number of studies reporting cluster	Factors
Structural and service delivery cluster	21	Accessibility
		Availability of services convenient to community members
		Household visits
		Perception of quality of health care
		Availability of medicine/drugs
		Availability of free services
		Availability of referral services from CHWs
Trust cluster	19	Trust in CHW
		Competency
		Confidentiality
		Familiarity
		Previous Experience
Identity and sociocultural cluster	16	Gender of CHW
		CHW from the same community
		Influence of partner/family

#### 3.5.1 Structural and Service Delivery Cluster

This thematic cluster encompasses the operational CHW service delivery. Its direct influence on community members' access to and experiences with health care makes it an important cluster. In essence, its structural components build essential infrastructure and mechanisms required for effective service delivery of CHW. Together, these factors determine if services are delivered to community members at the appropriate times and locations, with the required quality and thoroughness. The factors within this cluster are interconnected. Any deficiency or weakness in one component can negatively affect the perception of another factor.

##### 3.5.1.1 Accessibility to CHW

The importance of accessibility was underscored in ten studies in this review [61, 62, 65, 66, 68, 71, 74, 78, 81, 82]. Six of these studies demonstrated that easy access positively influenced community members' perceptions of CHW service utilisation [65, 66, 71, 74, 78, 82], while four studies found that difficult access had a negative impact [61, 62, 68, 81]. For instance, Shaw et al.'s study in Ethiopia revealed that households farther from CHW accessibility negatively affected the perception and community members were less likely to use the services [62], and Batte et al. found that long travel distances to receive CHW services negatively affected community members' perceptions [81]. Similarly, caregivers in another study in Ethiopia cited significant obstacles to accessing CHW services, including long distances, geographical barriers, inadequate transportation and challenges in transporting sick children, especially during the wet season, which created a negative perception of CHW services within the community [61].

##### 3.5.1.2 Availability of Services Convenient to Community Members

Besides accessibility as a factor affecting perception, the availability of services at times and locations convenient for community members was also identified as another significant factor influencing service utilisation. Eight studies support this finding [58, 61, 62, 65, 67, 74, 78, 82]. We found that individuals who perceived a health facility to be closed did not seek services due to negative perceptions arising from uncertainty regarding the availability of services [62]. However, the availability of services at convenient locations positively affects perception and enhances community members' willingness to utilise CHW's services [65, 74, 82].

##### 3.5.1.3 Household Visit by CHW

Some CHW programmes clearly designate a certain number of households for CHWs to provide services, and they visit each household a set number of times each month. These CHWs deliver services to people in the houses of community members [73]. Seven studies highlighted the significant impact of household visits by CHWs on community members' perceptions [55, 56, 70, 71, 72, 73, 78]. People were satisfied because they could continue their household tasks while receiving services from CHWs during home visits [70]. Research conducted in Kenya indicated that households receiving visits from CHWs were 1.8 times more likely to seek services from them [72].

##### 3.5.1.4 Perception of Quality of Healthcare

The quality of healthcare significantly shapes the community's perception of CHWs and their services. Our review identified five studies on this factor [62, 64, 74, 81, 82]. Yirgu et al. found that some participants indicated their preferred family planning service choices were not fulfilled by CHWs. In such cases, some women sought family planning services at private facilities to obtain their desired methods, particularly if they felt they could not access them through CHWs [64]. We also found that when community members perceive the quality of CHW services as inadequate, they do not use these services [62]; in some instances, they tend to seek healthcare from alternative providers [74].

##### 3.5.1.5 Availability of Medication With CHWs

The availability of medicines from CHWs and the provision of curative services by CHWs play roles in shaping community perceptions. We identified four studies that support this finding [62, 74, 79, 80]. Shaw et al. reported that the unavailability of medicine with CHWs (possibly due to supply chain issues) negatively affected community members' perception and use of services [62]. A study conducted in Tanzania indicated that CHWs were able to provide medications even when local health clinics and dispensaries lacked the necessary supplies. This availability led to an increased utilisation of services from CHWs. Community members commonly refer to these CHWs as “street doctors” and female CHWs as “street nurses,” highlighting their access to medications [79]. Similarly, a study conducted in Myanmar by Watt et al. found that perceived medication efficacy was associated with the quality of care. Participants often consider injections to be a superior form of treatment compared to oral medications [74].

##### 3.5.1.6 Availability of Free Services

This factor was identified by four studies [67, 71, 74, 75], which noted that free services increased the utilisation of services by CHWs.

##### 3.5.1.7 Availability of Referral Services From CHWs

There are two studies showing that referral services from CHWs influence the perception of community members and increase the use of the services of CHWs [74, 82].

#### 3.5.2 Trust Cluster

Factors within this thematic cluster are also interconnected. Competency of CHWs builds their credibility among community members. This strengthens their trust in CHWs. Similarly, confidentiality demonstrates respect and professionalism and reinforces trust in CHWs. Familiarity with CHWs creates comfort and rapport, facilitating the development of trust. Having a positive prior experience with CHWs also enhances trust in them. These relational elements operate synergistically, e.g., when a CHW shows technical competence and, in addition to having personal connections with community members, also shows confidentiality. This deepens the trust. Alternatively, breaches in any of these areas can diminish community members' trust in CHWs.

##### 3.5.2.1 Trust in CHWs

Trust in CHWs was identified as a factor influencing community members' perceptions of PHC services provided by CHWs in 10 studies [61, 64, 66, 71, 73, 74, 77, 80, 81, 82]. When trust is established, individuals begin to use the interventions offered by CHWs. It results in adherence to their guidance. However, in a study in Ethiopia, distrust due to ethnic conflicts led to reluctance to use government health programmes [61].

##### 3.5.2.2 Competency of CHWs

Seven studies found competency to be a factor influencing the perception of community members regarding PHC service delivery by CHWs [55, 64, 73, 74, 77, 82, 83]. From these studies, Grant et al. discovered that trusting relationships between CHWs and community members were associated with the perceived skills, knowledge and expertise of the CHWs [77]. In another study, Watt et al. identified experience and skills as key to establishing trust in Myanmar, while a lack of experience could lead to distrust [74]. Furthermore, Buchner et al., in their study in Uganda, found that uneducated CHWs and any misdiagnosis from them negatively affected the perception of community members [82].

##### 3.5.2.3 Confidentiality

We found that the perception of community members was also impacted by confidentiality. Our review uncovered seven studies supporting this finding [65, 66, 71, 77, 78, 84]. A South African study found that women initially expressed concerns about disclosing private information to CHWs. However, over time, they became more assured that their confidentiality was being maintained, which led to increased comfort in sharing personal information [78]. According to Stephen et al., women preferred seeking family planning services from CHWs because they trusted them to keep their visits confidential [66]. Confidentiality is important and it can change the perception of the community. Therefore, community members may seek services from CHWs in other areas if their local CHW fails to maintain confidentiality. Grant et al. reported that community members hesitated to share confidential information when they felt that CHWs were untrustworthy. Some opted to visit clinics in different locations to prevent being seen by local CHWs [77].

##### 3.5.2.4 Familiarity With CHWs

Although CHWs from the same community influence the perception of community members, familiarity with CHWs has also emerged as an important factor. It was highlighted in six studies [56, 65, 66, 67, 74, 77]. For instance, a study conducted in South Africa indicated that participants found it easier to establish a trusting relationship with someone they were familiar with [77]. Similarly, Watt et al. identified that personal relationships developed over time are essential for fostering trust between service users and providers [74].

##### 3.5.2.5 Previous Experience With CHWs

This systematic review also identified that previous experiences of users, as well as those of their family and friends with CHWs, significantly shape the perceptions of service users. We identified five studies that support this factor [58, 61, 62, 74, 81]. Community members reported that they relied on their own experiences and those of others when deciding whether to trust a healthcare provider or a particular medication [58, 74]. In a study conducted in Uganda, the trust built through the previous provision of various health services by CHWs was proven to be important [81].

#### 3.5.3 Identity and Sociocultural Cluster

This cluster comprises cultural, social and identity‐related factors that affect community members' perceptions of CHWs' service delivery. These factors are based on deeply rooted cultural values, social norms and community dynamics that influence health‐seeking behaviours and perception of community. One factor within this group, the Gender of CHWs, arises from cultural beliefs and norms. Additionally, community bonds enhance relationships through shared identity, language and experiences, thereby building trust and facilitating communication. Influences from family and peers illustrate the collective decision‐making processes often seen in many rural regions. The interrelation of these factors indicates that CHWs who reflect community identities and honour cultural norms related to gender and family relationships are more likely to be embraced and trusted.

##### 3.5.3.1 Gender of CHWs

Our study identified eight articles which showed that the gender of CHWs can significantly impact how community members perceive and accept the services they provide [57, 58, 65, 76, 77, 79, 83, 84]. For instance, one study found that a majority of women participants preferred services from female CHWs, particularly when discussing sensitive health issues [58]. Similarly, another study revealed that both men and women expressed a preference for CHWs of the same sex due to social and cultural norms [79]. Interestingly, a South African study has highlighted safety concerns regarding male CHWs [77]. On the other hand, younger women were found to be more comfortable discussing certain health issues with male CHWs, feeling less judged for their choices [79]. A number of community members in a study in South Africa expressed that the gender of the CHWs was irrelevant as long as they received proper training [77].

##### 3.5.3.2 CHW From the Same Community

Eight studies in our systematic review indicate that CHWs from the same community positively influence local perceptions regarding service utilisation [55, 58, 65, 75, 77, 78, 81, 83]. For example, participants in a study conducted in South Africa believed that CHWs from their own community had a deeper understanding of their circumstances. They were more approachable and would thus be more effective in their roles [77]. Similarly, research by Puett et al. in Bangladesh found that caretakers felt a strong mental connection with CHWs from their own community [55]. Furthermore, in a New Zealand study, participants reported that it was easier to talk to CHWs than to doctors. They felt more relaxed talking to CHWs, considering them as their own people. Participants also stated that CHWs treat them as part of the family. These experiences positively influenced the perception of community members and utilisation of health care services [75]. In Adamu's study, many community members shared that they began participating because they trusted the information from the CHWs. They view CHWs as “their people” who had a favourable attitude towards the community. They mentioned that when health information was delivered by “outsiders,” they were often sceptical. However, when the same health information was shared by CHWs, who are considered “insiders” in the community, they were more inclined to believe it [58]. Moreover, in Uganda, a study found that people sharing similar lifestyles with CHWs facilitated mutual understanding of each other's daily constraints. This resulted in fostering a more productive relationship [81]. However, in one study, some participants expressed worries about confidentiality and believed that a community health worker from an outside community would be more inclined to maintain the confidentiality of their information [77].

##### 3.5.3.3 Family and Peer Influence

This factor was observed in five studies [58, 61, 64, 71, 72]. A study from Ethiopia reported that lay reference networks of participants of the study affected their perception. The same study also revealed that elder family members influenced caregivers to consider herbal medicine before exploring other options [61]. In this regard, two studies highlighted that opposition from a partner regarding the use of services from CHWs negatively influenced perceptions of utilising these services [64, 71]. In contrast, one study found that the presence of a male partner at antenatal care (ANC) visits was positively associated with the utilisation of CHW services for treating fever in children under five [72].

## 4 Discussion

Numerous systematic reviews have been conducted on community health workers [7, 10, 26, 47, 88, 89, 90, 91, 92, 93, 94, 95]. To the best of our knowledge, this systematic review represents the first effort to comprehensively capture community perceptions and identify the factors that may positively or negatively affect community members' perceptions of primary health care (PHC) services delivered by community health workers (CHWs) in rural or remote areas. Even though there are significant differences between developing and developed nations, the main themes in rural health are consistent globally. Access remains the major challenge in rural health [96]. Most of the studies in our systematic review are from developing countries, which critically shapes the interpretation and generalisability, highlighting accessibility as a significant issue in these regions. CHWs either operate from health facilities or provide outreach services. Our findings indicate that individuals prefer receiving services at their own home through household visits by CHWs. Alternatively, the provision of services by CHWs at locations convenient for community members. This preference for services further underscores the existing accessibility challenges.

Our findings, which indicate that the availability of medications with CHWs, particularly injections, and the provision of curative services shape community perceptions, align with a systematic review of existing reviews on CHWs [47]. Furthermore, our observation that community involvement in the selection of CHWs positively alters perceptions of their services and that CHWs being from the same community enhances trust in their services is also aligned with the findings of this systematic review of existing reviews [47] as well as with another systematic review published in 2022 [88]. Grant et al. described that preserving confidentiality within closely connected communities can be difficult [77]. Yet it is crucial for fostering trust and ensuring effective use. Consequently, it is vital that the recruitment and training procedures for CHWs include clear confidentiality protocols and ethical standards, as endorsed by WHO best‐practice recommendations [97]. Balancing the benefits of local cultural congruence with safeguarding privacy represents a key tension in CHW programme design that must be actively managed through ongoing community and stakeholder engagement. According to Grant et al., some of the participants voiced concerns regarding confidentiality and felt that a CHW from a different community would be more likely to safeguard their information [77]. The systematic review by Scott et al. [47] shows that higher education levels in CHWs improve their effectiveness, reinforcing our evidence that CHWs' competency positively impacts community members' perceptions.

Moreover, our findings of the availability of services at places convenient to community members and free services are consistent with a systematic review by Ahmed et al. [88]. However, we encountered divergence regarding the impact of gender that differs from the conclusions of Ahmed et al. [88]. Additionally, our systematic review, in agreement with findings from Australian contexts [94], places special emphasis on the dual imperative. That is, CHWs must foster trust through sustained rapport and social familiarity yet must also safeguard professional boundaries and confidentiality. This requires ongoing refresher training to help CHWs manage their dual roles as insiders and outsiders while ensuring service quality and confidentiality amid close relationships.

A review of community health workers [93] revealed that socially disadvantaged patients expressed satisfaction with CHWs. In this regard, our findings regarding a lower educational level among community members led to a more positive perception of CHW services. Socially disadvantaged people with less education are more inclined towards utilising services from CHWs. It may be because less advantaged people may not have any other options for health care services. However, Juma et al. [69] suggest that individuals with higher education levels generally possess greater awareness of health issues. They are therefore more inclined to question the quality of family planning services provided by CHWs. This situation is opposite when services are provided by highly trained health professionals. Conversely, a separate study in Kenya indicated that increased utilisation of health services for child illness occurred in households where the head of the household had a secondary level of education [72]. This highlights the positive impact of education on perception. As higher‐quality services become accessible, increased educational levels among community members often encourage the utilisation of services from qualified healthcare professionals.

The three thematic clusters identified in this review (structural and service delivery, trust and identity and sociocultural) are interconnected and collectively influence how communities perceive and engage with PHC delivered by CHWs. The structural and service delivery cluster establishes the foundational element of effective provision of PHC by CHWs. This ensures their access, availability and quality of services. At the same time, the trust cluster emphasises the relational and experiential factors. Moreover, the identity and sociocultural cluster adds a contextual perspective. It influences both other thematic clusters. Together, these interdependent clusters demonstrate that enhancing CHW programmes necessitates a balanced approach that addresses logistical challenges, fosters trust‐based relationships and aligns service delivery with local cultural expectations to ensure equitable, person‐centred healthcare in rural areas.

The included studies in this review did not discuss language congruence. It may be because the CHWs in the included studies might have been local and shared the primary language with community members. This reflects the assumption that local CHWs share a common language with community members. However, within communities, there are often social diversities and the operation of CHWs in socially diverse communities requires further exploration. Additionally, in settings where CHWs are not local and speak a different language from community members, a lack of language congruence may arise as an issue.

This systematic review presents some limitations. In this review, our primary focus was on information sourced directly from community members. This led us to exclude studies that captured community perceptions reported by CHWs. This could have offered a valuable alternative perspective. Despite developing a comprehensive search strategy, it is possible that some studies were inadvertently overlooked. Furthermore, it is important to note that all quantitative studies included in this review are cross‐sectional, with none being longitudinal in nature.

This review offers important perspectives that could greatly influence multiple practical fields. Tailoring training to build trust and competence in culturally sensitive ways can help CHWs address community challenges, reduce stigma around health services and promote confidentiality. The potential for policy applications in this area is significant. The review highlights the necessity for health systems to concentrate on key factors by improving the ability of CHWs to provide reliable, high‐quality care, easing accessibility issues and designing CHW programmes responsive to the needs of the community. By doing so, health systems can build greater trust within the community.

Additionally, the findings of this review may inform a more evidence‐based approach to supervising CHWs. Supervisors can utilise the findings regarding factors that influence perceptions of community members to assist CHWs. It will ensure that they appropriately address the unique issues of the communities they deliver PHC services. By acknowledging and acting on these factors, CHW programmes, or individual CHWs, can establish a strong foundation for a trusting relationship with community members. This encourages participation and utilisation, aligns services with people's needs and values. Furthermore, establishing trust, providing quality services and respecting cultural values will encourage community members to utilise CHWs' services.

Further research needs to be conducted on the perceptions of community members from the perspective of CHWs. This would highlight and identify the factors that CHWs have observed during their experiences. There is a need for research on how to improve the community perception using a longitudinal study.

## 5 Conclusion

This systematic review identified several factors influencing community perceptions of primary health care (PHC) services delivered by community health workers (CHWs) in rural areas. These factors can guide policymakers in developing community‐focused PHC strategies. Such strategies should not only prioritise structural and service delivery aspects but also foster community trust and respect local cultural contexts. Incorporating these findings into the CHW training curriculum would better prepare CHWs to meet community expectations. At the grassroots level, CHWs and their supervisors can use this evidence to adapt services more effectively to service recipients' needs. This approach will enhance service quality, align health care with local culture and foster trust‐based relationships with rural communities. In conclusion, this systematic review identified important factors for enhancing CHWs' delivery of PHC services in rural areas and ensuring these services are sustainable and responsive to community needs.

## Author Contributions

**Niaz Ahmed:** conceptualisation (lead), data curation (lead), formal analysis (lead), investigation (lead), methodology (lead), project administration (lead), resources (lead), software (lead), validation (lead), visualisation (lead), writing – original draft preparation (lead), writing – review and editing (lead). **Maginsh Dahal:** data curation (equal), formal analysis (equal), investigation (equal), software (equal). **Hamish Crocket:** supervision, conceptualisation (support), data curation (support), investigation (support), methodology (support), validation (support), writing – review and editing (support). **Roger Strasser:** supervision, conceptualisation (support), data curation (support), investigation (support), methodology (support), validation (support), writing – review and editing (support).

## Funding

The authors have nothing to report.

## Conflicts of Interest

The authors declare no conflicts of interest.

## Supporting information


Supporting Information S1.

Supporting Information S2.

## Data Availability

Data sharing not applicable to this article as no datasets were generated or analysed during the current study.
