# Supplementary material for: Factors Influencing Community Members' Perception of Primary Health Care Services Delivered by Community Health Workers in Rural Areas: A Systematic Review
Source: Aust J Rural Health. 2026 Jan 19;34(1):e70142. doi: 10.1111/ajr.70142 (PMC12921660; doi:10.1111/ajr.70142)
Supplement: Supplementary file 1 — Supporting Information S1. [file AJR-34-0-s001.docx]

|  | | **Supplementary Material 1**  **Search strategy for included databases (PubMed, Scopus, Ovid Medline, ProQuest Central, CINHAL, Cochrane Library and APA PsycINFO)**  **Summary of Database Search** | | | | | | | | | | | | | | | | | | | | | |
| --- | --- | --- | --- | --- | --- | --- | --- | --- | --- | --- | --- | --- | --- | --- | --- | --- | --- | --- | --- | --- | --- | --- | --- |
|  | | **S.#** | | **Database** | | **Initial Search Results** | | **Search results after applying filters**† | | **Duplicate articles detected by EndNote** | | **Duplicates detected by Rayyan** | | **Manual Deduplication** | | **Total Duplicates removed** | | **Number of articles reviewed for Titles and Abstracts** | | **Number of articles eligible for full-text Review** | | **Number of articles included in the study** | |
|  | | 1 | | PubMed | | 2557 | | 2236 | | 1492 | | 381 | | 4 | | 1877 | | 3119 | | 231 | | 30 | |
|  | | 2 | | Scopus | | 1043 | | 1009 | |  |  |  |  |  |  |  |  |  |  |  |  |  |  |
|  | | 3 | | Ovid Medline | | 657 | | 547 | |  |  |  |  |  |  |  |  |  |  |  |  |  |  |
|  | | 4 | | ProQuest Central | | 561 | | 558 | |  |  |  |  |  |  |  |  |  |  |  |  |  |  |
|  | | 5 | | CINAHL | | 366 | | 360 | |  |  |  |  |  |  |  |  |  |  |  |  |  |  |
|  | | 6 | | Cochrane Library | | 239 | | 239 | |  |  |  |  |  |  |  |  |  |  |  |  |  |  |
|  | | 7 | | APA PsycINFO (OVID) | | 48 | | 47 | |  |  |  |  |  |  |  |  |  |  |  |  |  |  |
|  | |  | | Total | | 5471 | | 4996 | |  |  |  |  |  |  |  |  |  |  |  |  |  |  |
|  | | †Filters:Language: English, Time Duration: 1990-2024 | | | | | | | | | | | | | | | | | | | | | |
|  | |  | |  | |  | |  | |  | |  | |  | |  | |  | |  | |  | |
|  | |  | |  | | | | | | | | | | | | | | | |  | |  | |

**Search Strategy for PubMed**

| **Search #** | **Field** | **keyword** | **Results** |
| --- | --- | --- | --- |
| **1** | Title/Abstract | “Health auxiliary” OR “frontline health workers” OR “frontline health worker” OR midwife OR Midwifery OR midwives OR “Birth Attendant” OR Midwives OR “outreach worker” OR “outreach workers” OR “lay health worker” OR “lay health workers” OR promotora OR promotoras OR “village health worker” OR “village health workers” OR “volunteer health worker” OR “volunteer health workers” OR “voluntary health workers” OR “voluntary health worker” OR “community health agent” OR “community health agents” OR “health promoter” OR “health promoters” OR “community health worker” OR “community health workers” OR “community health aide” OR “community health aides” OR “community health nursing” OR “community health nurses” OR “community health nurse” OR “community health officers” OR “community health officer” OR “community health volunteer” OR “community health volunteers” OR “community health distributors” OR “community health distributor” OR “community health surveyors” OR “community health surveyor” OR “community health assistants” OR “community health assistant” OR “community health promoters” OR “community health promoters” OR “community IMCI” OR “community volunteer” OR “community volunteers” OR “health extension workers” OR “health extension worker” OR “village health volunteer” OR “village health volunteers” OR “close-to-community provider” OR “close-to-community providers” OR “community-based practitioner” OR “community-based practitioners” OR “lady Health worker” OR “lady Health workers” OR “barefoot doctor” OR “Community Practitioners” OR “Community Practitioner” OR “community-based practitioners” OR “community-based practitioner” OR “promotoras de salud” OR “agentes de saúde” OR “rural health auxiliaries” OR “traditional birth attendants” OR “traditional birth attendant” OR Activista OR “Agente comunitario de salud” OR “Agente comunitário de saúde” OR Anganwadi OR Animatrice OR “Barangay health worker” OR “Barangay health workers” OR “Basic health worker” OR “Basic health workers” OR Brigadista OR “Colaborador voluntario” OR “Community drug distributor” OR “Community drug distributors” OR “Community health agent” OR “Community health agents” OR “Community health promoter” OR “Community health promoters” OR “Community health representative” OR “Community health representatives” OR “Community health volunteer” OR “Community health volunteers” OR “Community resource person” OR “Female multipurpose health worker” OR “Female multipurpose health worker” OR “Health promoter” OR “Health promoters” OR Kader OR Monitora OR “Mother coordinator” OR “Outreach educator” OR “Outreach educators” OR Promotora OR “Shastho shebika” OR “Shastho karmis” OR Sevika OR “Village health helper” OR “Village drug-kit manager” OR Accompagnateur OR “Accredited Social Health Activist” OR Animator OR ASHA OR “Auxiliary Nurse” OR “Auxiliary Nurse-midwife” OR “Bridge-to-Health Team” OR Behvarz OR “Care Group” OR “Care Groups” OR “Care Group Volunteer” OR “Care Group Volunteers” OR “Community Case Management Worker” OR “Community Case Management Workers” OR “Community Health Agent” OR “Community Health Agents” OR “Community Health Care Provider” OR “Community Health Care Providers” OR “Community HealthCare Provider” OR “Community HealthCare Providers” OR “Community Health Extension Worker” OR “Community Health Extension Workers” OR “Community Health Officer” OR “Community Health Officers” OR “Community Surveillance Volunteer” OR “Community Surveillance Volunteers” OR “Family Health Worker” OR “Family Health Workers” OR “Family Planning Agent” OR “Family Planning Agents” OR “Family Welfare Assistant” OR “Family Welfare Assistants” OR “Female Community Health Volunteer” OR “Female Community Health Volunteers” OR “Health Agent” OR “Health Agents” OR “Health Assistant” OR “Health Assistants” OR “Health Extension Worker” OR “Health Extension Workers” OR “Health Surveillance Assistant” OR “Health Surveillance Assistants” OR “Lead Mother” OR “Malaria Agent” OR “Malaria Agents” OR “Maternal and Child Health Worker” OR “Maternal and Child Health Workers” OR “Mobile Clinic Team” OR “Mobile Clinic Teams” OR “Nutrition Agent” OR “Nutrition Agents” OR “Nutrition Counselor” OR “Nutrition Counselors” OR “Peer Educator” OR “Peer Educators” OR “Shasthya Shebika” OR Socorrista |  |
| **2** | MeSH Terms | Community Health Workers |  |
| **3** |  | S1 OR S2 | 60679 |
| **4** | Title/Abstract | "Primary Health Care" OR "Primary Healthcare” OR “Primary Care” OR “Comprehensive Primary Health Care” OR “Selective Primary Health Care” OR “Vertical Programme” OR "Vertical programmes" OR "Vertical Program" OR "Vertical Programs" OR "Community Health Services" |  |
| **5** | MeSH Terms | primary health care OR Community health services |  |
| **6** |  | S4 OR S5 | 616281 |
| **7** | Title/abstract | Perception* OR "community perception*" OR "patient perception*" OR perspective OR "Community perspective" OR "patient perspective" OR attitudes OR "attitude to health" OR opinion* OR "opinion of community" OR "opinion of patient" OR feeling* OR "community feelings" OR "feeling of patient" OR experience* OR "community experience" OR " patient experience" OR satisfaction* OR "community satisfaction*" OR "patient satisfaction" OR acceptance* OR "community acceptance*" OR "patient acceptance*" OR "patient acceptance of health care" OR uptake OR utilisation* OR "service utilisation*" OR "community utilisation*" OR "patient utilisation*" OR barrier OR "barrier to health" OR "attitude to health" OR "quality of health care" |  |
| **8** | MeSH Terms | attitude to health OR patient satisfaction OR patient acceptance of health care OR quality of health care |  |
| **9** |  | S7 OR S8 | 10416550 |
| **10** | Title/abstract | Remote OR "remote area" OR "remote areas" OR "remote region" OR "remote regions" OR rural OR "rural area" OR "rural areas" OR "rural health service" OR "rural health services" OR "Rural Health" OR "rural health center" OR "rural health centers" OR "rural health centre" OR "rural health centres" OR "rural population" OR "rural populations" OR "rural community" OR "rural communities" OR "rural catchments" OR "rural catchment" |  |
| **11** | MeSH Terms | Rural population OR Rural health services OR rural health |  |
| **12** |  | S10 OR S11 | 316478 |
| **13** |  | S1 (OR S2) AND S4 (OR S5) AND S7 (OR S8) AND S10 (OR S11) | 2557 |
| **14** | After applying filters | Filters: English Language  Duration:1990-2024 | 2236 |

**Search Strategy for OVID Medline**

| **Search #** | **Field** | **keyword** | **Results** |
| --- | --- | --- | --- |
| **1** | Title | “Health auxiliary” OR “frontline health workers” OR “frontline health worker” OR midwife OR Midwifery OR midwives OR “Birth Attendant” OR Midwives OR “outreach worker” OR “outreach workers” OR “lay health worker” OR “lay health workers” OR promotora OR promotoras OR “village health worker” OR “village health workers” OR “volunteer health worker” OR “volunteer health workers” OR “voluntary health workers” OR “voluntary health worker” OR “community health agent” OR “community health agents” OR “health promoter” OR “health promoters” OR “community health worker” OR “community health workers” OR “community health aide” OR “community health aides” OR “community health nursing” OR “community health nurses” OR “community health nurse” OR “community health officers” OR “community health officer” OR “community health volunteer” OR “community health volunteers” OR “community health distributors” OR “community health distributor” OR “community health surveyors” OR “community health surveyor” OR “community health assistants” OR “community health assistant” OR “community health promoters” OR “community health promoters” OR “community IMCI” OR “community volunteer” OR “community volunteers” OR “health extension workers” OR “health extension worker” OR “village health volunteer” OR “village health volunteers” OR “close-to-community provider” OR “close-to-community providers” OR “community-based practitioner” OR “community-based practitioners” OR “lady Health worker” OR “lady Health workers” OR “barefoot doctor” OR “Community Practitioners” OR “Community Practitioner” OR “community-based practitioners” OR “community-based practitioner” OR “promotoras de salud” OR “agentes de saúde” OR “rural health auxiliaries” OR “traditional birth attendants” OR “traditional birth attendant” OR Activista OR “Agente comunitario de salud” OR “Agente comunitário de saúde” OR Anganwadi OR Animatrice OR “Barangay health worker” OR “Barangay health workers” OR “Basic health worker” OR “Basic health workers” OR Brigadista OR “Colaborador voluntario” OR “Community drug distributor” OR “Community drug distributors” OR “Community health agent” OR “Community health agents” OR “Community health promoter” OR “Community health promoters” OR “Community health representative” OR “Community health representatives” OR “Community health volunteer” OR “Community health volunteers” OR “Community resource person” OR “Female multipurpose health worker” OR “Female multipurpose health worker” OR “Health promoter” OR “Health promoters” OR Kader OR Monitora OR “Mother coordinator” OR “Outreach educator” OR “Outreach educators” OR Promotora OR “Shastho shebika” OR “Shastho karmis” OR Sevika OR “Village health helper” OR “Village drug-kit manager” OR Accompagnateur OR “Accredited Social Health Activist” OR Animator OR ASHA OR “Auxiliary Nurse” OR “Auxiliary Nurse-midwife” OR “Bridge-to-Health Team” OR Behvarz OR “Care Group” OR “Care Groups” OR “Care Group Volunteer” OR “Care Group Volunteers” OR “Community Case Management Worker” OR “Community Case Management Workers” OR “Community Health Agent” OR “Community Health Agents” OR “Community Health Care Provider” OR “Community Health Care Providers” OR “Community HealthCare Provider” OR “Community HealthCare Providers” OR “Community Health Extension Worker” OR “Community Health Extension Workers” OR “Community Health Officer” OR “Community Health Officers” OR “Community Surveillance Volunteer” OR “Community Surveillance Volunteers” OR “Family Health Worker” OR “Family Health Workers” OR “Family Planning Agent” OR “Family Planning Agents” OR “Family Welfare Assistant” OR “Family Welfare Assistants” OR “Female Community Health Volunteer” OR “Female Community Health Volunteers” OR “Health Agent” OR “Health Agents” OR “Health Assistant” OR “Health Assistants” OR “Health Extension Worker” OR “Health Extension Workers” OR “Health Surveillance Assistant” OR “Health Surveillance Assistants” OR “Lead Mother” OR “Malaria Agent” OR “Malaria Agents” OR “Maternal and Child Health Worker” OR “Maternal and Child Health Workers” OR “Mobile Clinic Team” OR “Mobile Clinic Teams” OR “Nutrition Agent” OR “Nutrition Agents” OR “Nutrition Counselor” OR “Nutrition Counselors” OR “Peer Educator” OR “Peer Educators” OR “Shasthya Shebika” OR Socorrista |  |
| **2** | Abstract | “Health auxiliary” OR “frontline health workers” OR “frontline health worker” OR midwife OR Midwifery OR midwives OR “Birth Attendant” OR Midwives OR “outreach worker” OR “outreach workers” OR “lay health worker” OR “lay health workers” OR promotora OR promotoras OR “village health worker” OR “village health workers” OR “volunteer health worker” OR “volunteer health workers” OR “voluntary health workers” OR “voluntary health worker” OR “community health agent” OR “community health agents” OR “health promoter” OR “health promoters” OR “community health worker” OR “community health workers” OR “community health aide” OR “community health aides” OR “community health nursing” OR “community health nurses” OR “community health nurse” OR “community health officers” OR “community health officer” OR “community health volunteer” OR “community health volunteers” OR “community health distributors” OR “community health distributor” OR “community health surveyors” OR “community health surveyor” OR “community health assistants” OR “community health assistant” OR “community health promoters” OR “community health promoters” OR “community IMCI” OR “community volunteer” OR “community volunteers” OR “health extension workers” OR “health extension worker” OR “village health volunteer” OR “village health volunteers” OR “close-to-community provider” OR “close-to-community providers” OR “community-based practitioner” OR “community-based practitioners” OR “lady Health worker” OR “lady Health workers” OR “barefoot doctor” OR “Community Practitioners” OR “Community Practitioner” OR “community-based practitioners” OR “community-based practitioner” OR “promotoras de salud” OR “agentes de saúde” OR “rural health auxiliaries” OR “traditional birth attendants” OR “traditional birth attendant” OR Activista OR “Agente comunitario de salud” OR “Agente comunitário de saúde” OR Anganwadi OR Animatrice OR “Barangay health worker” OR “Barangay health workers” OR “Basic health worker” OR “Basic health workers” OR Brigadista OR “Colaborador voluntario” OR “Community drug distributor” OR “Community drug distributors” OR “Community health agent” OR “Community health agents” OR “Community health promoter” OR “Community health promoters” OR “Community health representative” OR “Community health representatives” OR “Community health volunteer” OR “Community health volunteers” OR “Community resource person” OR “Female multipurpose health worker” OR “Female multipurpose health worker” OR “Health promoter” OR “Health promoters” OR Kader OR Monitora OR “Mother coordinator” OR “Outreach educator” OR “Outreach educators” OR Promotora OR “Shastho shebika” OR “Shastho karmis” OR Sevika OR “Village health helper” OR “Village drug-kit manager” OR Accompagnateur OR “Accredited Social Health Activist” OR Animator OR ASHA OR “Auxiliary Nurse” OR “Auxiliary Nurse-midwife” OR “Bridge-to-Health Team” OR Behvarz OR “Care Group” OR “Care Groups” OR “Care Group Volunteer” OR “Care Group Volunteers” OR “Community Case Management Worker” OR “Community Case Management Workers” OR “Community Health Agent” OR “Community Health Agents” OR “Community Health Care Provider” OR “Community Health Care Providers” OR “Community HealthCare Provider” OR “Community HealthCare Providers” OR “Community Health Extension Worker” OR “Community Health Extension Workers” OR “Community Health Officer” OR “Community Health Officers” OR “Community Surveillance Volunteer” OR “Community Surveillance Volunteers” OR “Family Health Worker” OR “Family Health Workers” OR “Family Planning Agent” OR “Family Planning Agents” OR “Family Welfare Assistant” OR “Family Welfare Assistants” OR “Female Community Health Volunteer” OR “Female Community Health Volunteers” OR “Health Agent” OR “Health Agents” OR “Health Assistant” OR “Health Assistants” OR “Health Extension Worker” OR “Health Extension Workers” OR “Health Surveillance Assistant” OR “Health Surveillance Assistants” OR “Lead Mother” OR “Malaria Agent” OR “Malaria Agents” OR “Maternal and Child Health Worker” OR “Maternal and Child Health Workers” OR “Mobile Clinic Team” OR “Mobile Clinic Teams” OR “Nutrition Agent” OR “Nutrition Agents” OR “Nutrition Counselor” OR “Nutrition Counselors” OR “Peer Educator” OR “Peer Educators” OR “Shasthya Shebika” OR Socorrista |  |
| **3** | MeSH subject Heading | Community Health Workers |  |
| **4** |  | S1 OR S2 OR S3 | 57879 |
| **5** | Title | "Primary Health Care" OR "Primary Healthcare” OR “Primary Care” OR “Comprehensive Primary Health Care” OR “Selective Primary Health Care” OR “Vertical Programme” OR "Vertical programmes" OR "Vertical Program" OR "Vertical Programs" OR "Community Health Services" |  |
| **6** | Abstract | "Primary Health Care" OR "Primary Healthcare” OR “Primary Care” OR “Comprehensive Primary Health Care” OR “Selective Primary Health Care” OR “Vertical Programme” OR "Vertical programmes" OR "Vertical Program" OR "Vertical Programs" OR "Community Health Services" |  |
| **7** | MeSH subject Heading | primary health care OR Community health services |  |
| **8** |  | S5 OR S6 ORS7 | 231182 |
| **9** | Title | Perception* OR "community perception*" OR "patient perception*" OR perspective OR "Community perspective" OR "patient perspective" OR attitudes OR "attitude to health" OR opinion* OR "opinion of community" OR "opinion of patient" OR feeling* OR "community feelings" OR "feeling of patient" OR experience* OR "community experience" OR " patient experience" OR satisfaction* OR "community satisfaction*" OR "patient satisfaction" OR acceptance* OR "community acceptance*" OR "patient acceptance*" OR "patient acceptance of health care" OR uptake OR utilisation* OR "service utilisation*" OR "community utilisation*" OR "patient utilisation*" OR barrier OR "barrier to health" OR "attitude to health" OR "quality of health care" |  |
| **10** | Abstract | Perception* OR "community perception*" OR "patient perception*" OR perspective OR "Community perspective" OR "patient perspective" OR attitudes OR "attitude to health" OR opinion* OR "opinion of community" OR "opinion of patient" OR feeling* OR "community feelings" OR "feeling of patient" OR experience* OR "community experience" OR " patient experience" OR satisfaction* OR "community satisfaction*" OR "patient satisfaction" OR acceptance* OR "community acceptance*" OR "patient acceptance*" OR "patient acceptance of health care" OR uptake OR utilisation* OR "service utilisation*" OR "community utilisation*" OR "patient utilisation*" OR barrier OR "barrier to health" OR "attitude to health" OR "quality of health care" |  |
| **11** | MeSH subject Heading | attitude to health OR patient satisfaction OR patient acceptance of health care OR quality of health care |  |
| **12** |  | S9 OR S10 OR S11 | 3244207 |
| **13** | Title | Remote OR "remote area" OR "remote areas" OR "remote region" OR "remote regions" OR rural OR "rural area" OR "rural areas" OR "rural health service" OR "rural health services" OR "Rural Health" OR "rural health center" OR "rural health centers" OR "rural health centre" OR "rural health centres" OR "rural population" OR "rural populations" OR "rural community" OR "rural communities" OR "rural catchments" OR "rural catchment" |  |
| **14** | Abstract | Remote OR "remote area" OR "remote areas" OR "remote region" OR "remote regions" OR rural OR "rural area" OR "rural areas" OR "rural health service" OR "rural health services" OR "Rural Health" OR "rural health center" OR "rural health centers" OR "rural health centre" OR "rural health centres" OR "rural population" OR "rural populations" OR "rural community" OR "rural communities" OR "rural catchments" OR "rural catchment" |  |
| **15** | MeSH subject Heading | Rural population OR Rural health services OR rural health |  |
| **16** |  | S13 OR S14 ORS15 | 304423 |
| **17** |  | S4 AND S8 AND S12 AND S16 | 657 |
| **18** | After applying filters | Filters: English Language  Duration:1990-2024 | 547 |

**Search Strategy for Scopus**

| **Search #** | **Field** | **keyword** | **Results** |
| --- | --- | --- | --- |
| **1** | Title/Abstract | “Health auxiliary” OR “frontline health workers” OR “frontline health worker” OR midwife OR Midwifery OR midwives OR “Birth Attendant” OR Midwives OR “outreach worker” OR “outreach workers” OR “lay health worker” OR “lay health workers” OR promotora OR promotoras OR “village health worker” OR “village health workers” OR “volunteer health worker” OR “volunteer health workers” OR “voluntary health workers” OR “voluntary health worker” OR “community health agent” OR “community health agents” OR “health promoter” OR “health promoters” OR “community health worker” OR “community health workers” OR “community health aide” OR “community health aides” OR “community health nursing” OR “community health nurses” OR “community health nurse” OR “community health officers” OR “community health officer” OR “community health volunteer” OR “community health volunteers” OR “community health distributors” OR “community health distributor” OR “community health surveyors” OR “community health surveyor” OR “community health assistants” OR “community health assistant” OR “community health promoters” OR “community health promoters” OR “community IMCI” OR “community volunteer” OR “community volunteers” OR “health extension workers” OR “health extension worker” OR “village health volunteer” OR “village health volunteers” OR “close-to-community provider” OR “close-to-community providers” OR “community-based practitioner” OR “community-based practitioners” OR “lady Health worker” OR “lady Health workers” OR “barefoot doctor” OR “Community Practitioners” OR “Community Practitioner” OR “community-based practitioners” OR “community-based practitioner” OR “promotoras de salud” OR “agentes de saúde” OR “rural health auxiliaries” OR “traditional birth attendants” OR “traditional birth attendant” OR Activista OR “Agente comunitario de salud” OR “Agente comunitário de saúde” OR Anganwadi OR Animatrice OR “Barangay health worker” OR “Barangay health workers” OR “Basic health worker” OR “Basic health workers” OR Brigadista OR “Colaborador voluntario” OR “Community drug distributor” OR “Community drug distributors” OR “Community health agent” OR “Community health agents” OR “Community health promoter” OR “Community health promoters” OR “Community health representative” OR “Community health representatives” OR “Community health volunteer” OR “Community health volunteers” OR “Community resource person” OR “Female multipurpose health worker” OR “Female multipurpose health worker” OR “Health promoter” OR “Health promoters” OR Kader OR Monitora OR “Mother coordinator” OR “Outreach educator” OR “Outreach educators” OR Promotora OR “Shastho shebika” OR “Shastho karmis” OR Sevika OR “Village health helper” OR “Village drug-kit manager” OR Accompagnateur OR “Accredited Social Health Activist” OR Animator OR ASHA OR “Auxiliary Nurse” OR “Auxiliary Nurse-midwife” OR “Bridge-to-Health Team” OR Behvarz OR “Care Group” OR “Care Groups” OR “Care Group Volunteer” OR “Care Group Volunteers” OR “Community Case Management Worker” OR “Community Case Management Workers” OR “Community Health Agent” OR “Community Health Agents” OR “Community Health Care Provider” OR “Community Health Care Providers” OR “Community HealthCare Provider” OR “Community HealthCare Providers” OR “Community Health Extension Worker” OR “Community Health Extension Workers” OR “Community Health Officer” OR “Community Health Officers” OR “Community Surveillance Volunteer” OR “Community Surveillance Volunteers” OR “Family Health Worker” OR “Family Health Workers” OR “Family Planning Agent” OR “Family Planning Agents” OR “Family Welfare Assistant” OR “Family Welfare Assistants” OR “Female Community Health Volunteer” OR “Female Community Health Volunteers” OR “Health Agent” OR “Health Agents” OR “Health Assistant” OR “Health Assistants” OR “Health Extension Worker” OR “Health Extension Workers” OR “Health Surveillance Assistant” OR “Health Surveillance Assistants” OR “Lead Mother” OR “Malaria Agent” OR “Malaria Agents” OR “Maternal and Child Health Worker” OR “Maternal and Child Health Workers” OR “Mobile Clinic Team” OR “Mobile Clinic Teams” OR “Nutrition Agent” OR “Nutrition Agents” OR “Nutrition Counselor” OR “Nutrition Counselors” OR “Peer Educator” OR “Peer Educators” OR “Shasthya Shebika” OR Socorrista | 116109 |
| **2** | Title/Abstract | "Primary Health Care" OR "Primary Healthcare” OR “Primary Care” OR “Comprehensive Primary Health Care” OR “Selective Primary Health Care” OR “Vertical Programme” OR "Vertical programmes" OR "Vertical Program" OR "Vertical Programs" OR "Community Health Services" | 284190 |
| **3** | Title/abstract | Perception* OR "community perception*" OR "patient perception*" OR perspective OR "Community perspective" OR "patient perspective" OR attitudes OR "attitude to health" OR opinion* OR "opinion of community" OR "opinion of patient" OR feeling* OR "community feelings" OR "feeling of patient" OR experience* OR "community experience" OR " patient experience" OR satisfaction* OR "community satisfaction*" OR "patient satisfaction" OR acceptance* OR "community acceptance*" OR "patient acceptance*" OR "patient acceptance of health care" OR uptake OR utilisation* OR "service utilisation*" OR "community utilisation*" OR "patient utilisation*" OR barrier OR "barrier to health" OR "attitude to health" OR "quality of health care" | 9,275,523 |
| **4** | Title/abstract | Remote OR "remote area" OR "remote areas" OR "remote region" OR "remote regions" OR rural OR "rural area" OR "rural areas" OR "rural health service" OR "rural health services" OR "Rural Health" OR "rural health center" OR "rural health centers" OR "rural health centre" OR "rural health centres" OR "rural population" OR "rural populations" OR "rural community" OR "rural communities" OR "rural catchments" OR "rural catchment" | 1213887 |
| **5** |  | S1 AND S2 AND S3 AND S4 | 1043 |
| **6** | After applying Filters | Filters (Duration: 1990- 2024, Language: English) | 1009 |

**Search Strategy for Cochrane Library**

| **Search #** | **Field** | **keyword** | **Results** |
| --- | --- | --- | --- |
| **1** | Title/abstract | “Community Health Workers” OR "Lay Health Workers" OR "Health Auxiliary" OR "Lady Health Workers" OR "voluntary health workers" OR "health extension workers" OR behvarz OR "community health extension workers" OR "village health workers" |  |
| **2** | Title/abstract | Primary Health Care OR “Primary Care” OR “Comprehensive Primary Health Care” OR “Selective Primary Health Care” OR “Vertical Programme” OR "Vertical Program" OR "Community Health Services" |  |
| **3** | Title/abstract | Perception* OR (community NEXT perception*) OR (patient NEXT perception*) OR perspective OR "Community perspective" OR "patient perspective" OR attitudes OR "attitude to health" OR opinion* OR "opinion of community" OR "opinion of patient" OR feeling* OR "community feelings" OR "feeling of patient" OR experience* OR "community experience" OR " patient experience" OR satisfaction* OR (community NEXT satisfaction*) OR "patient satisfaction" OR acceptance* OR (community NEXT acceptance*) OR (patient NEXT acceptance*) OR "patient acceptance of health care" OR uptake OR utilisation* OR (service NEXT utilisation*) OR (community NEXT utilisation* ) OR (patient NEXT utilisation*) OR barrier OR "barrier to health" OR "attitude to health" OR "quality of health care" |  |
| **4** | Title/abstract | Remote OR "remote areas" OR "remote regions" OR rural OR "rural areas" OR "rural health services" OR "rural health centers" OR "rural health centres" OR "rural population" OR "rural populations" OR "rural community" OR "rural communities" OR "rural catchments" |  |
| **5** |  | S1 AND S2 AND S3 AND S4 | 239 |
| **6** | After applying filters | Filters (Duration: 1990- 2024, Language: English) | 239 |

**Search Strategy for ProQuest Central**

| **Search #** | **Field** | **keyword** | **Results** |
| --- | --- | --- | --- |
| **1** | abstract | “Health auxiliary” OR “frontline health workers” OR “frontline health worker” OR midwife OR Midwifery OR midwives OR “Birth Attendant” OR Midwives OR “outreach worker” OR “outreach workers” OR “lay health worker” OR “lay health workers” OR promotora OR promotoras OR “village health worker” OR “village health workers” OR “volunteer health worker” OR “volunteer health workers” OR “voluntary health workers” OR “voluntary health worker” OR “community health agent” OR “community health agents” OR “health promoter” OR “health promoters” OR “community health worker” OR “community health workers” OR “community health aide” OR “community health aides” OR “community health nursing” OR “community health nurses” OR “community health nurse” OR “community health officers” OR “community health officer” OR “community health volunteer” OR “community health volunteers” OR “community health distributors” OR “community health distributor” OR “community health surveyors” OR “community health surveyor” OR “community health assistants” OR “community health assistant” OR “community health promoters” OR “community health promoters” OR “community IMCI” OR “community volunteer” OR “community volunteers” OR “health extension workers” OR “health extension worker” OR “village health volunteer” OR “village health volunteers” OR “close-to-community provider” OR “close-to-community providers” OR “community-based practitioner” OR “community-based practitioners” OR “lady Health worker” OR “lady Health workers” OR “barefoot doctor” OR “Community Practitioners” OR “Community Practitioner” OR “community-based practitioners” OR “community-based practitioner” OR “promotoras de salud” OR “agentes de saúde” OR “rural health auxiliaries” OR “traditional birth attendants” OR “traditional birth attendant” OR Activista OR “Agente comunitario de salud” OR “Agente comunitário de saúde” OR Anganwadi OR Animatrice OR “Barangay health worker” OR “Barangay health workers” OR “Basic health worker” OR “Basic health workers” OR Brigadista OR “Colaborador voluntario” OR “Community drug distributor” OR “Community drug distributors” OR “Community health agent” OR “Community health agents” OR “Community health promoter” OR “Community health promoters” OR “Community health representative” OR “Community health representatives” OR “Community health volunteer” OR “Community health volunteers” OR “Community resource person” OR “Female multipurpose health worker” OR “Female multipurpose health worker” OR “Health promoter” OR “Health promoters” OR Kader OR Monitora OR “Mother coordinator” OR “Outreach educator” OR “Outreach educators” OR Promotora OR “Shastho shebika” OR “Shastho karmis” OR Sevika OR “Village health helper” OR “Village drug-kit manager” OR Accompagnateur OR “Accredited Social Health Activist” OR Animator OR ASHA OR “Auxiliary Nurse” OR “Auxiliary Nurse-midwife” OR “Bridge-to-Health Team” OR Behvarz OR “Care Group” OR “Care Groups” OR “Care Group Volunteer” OR “Care Group Volunteers” OR “Community Case Management Worker” OR “Community Case Management Workers” OR “Community Health Agent” OR “Community Health Agents” OR “Community Health Care Provider” OR “Community Health Care Providers” OR “Community HealthCare Provider” OR “Community HealthCare Providers” OR “Community Health Extension Worker” OR “Community Health Extension Workers” OR “Community Health Officer” OR “Community Health Officers” OR “Community Surveillance Volunteer” OR “Community Surveillance Volunteers” OR “Family Health Worker” OR “Family Health Workers” OR “Family Planning Agent” OR “Family Planning Agents” OR “Family Welfare Assistant” OR “Family Welfare Assistants” OR “Female Community Health Volunteer” OR “Female Community Health Volunteers” OR “Health Agent” OR “Health Agents” OR “Health Assistant” OR “Health Assistants” OR “Health Extension Worker” OR “Health Extension Workers” OR “Health Surveillance Assistant” OR “Health Surveillance Assistants” OR “Lead Mother” OR “Malaria Agent” OR “Malaria Agents” OR “Maternal and Child Health Worker” OR “Maternal and Child Health Workers” OR “Mobile Clinic Team” OR “Mobile Clinic Teams” OR “Nutrition Agent” OR “Nutrition Agents” OR “Nutrition Counselor” OR “Nutrition Counselors” OR “Peer Educator” OR “Peer Educators” OR “Shasthya Shebika” OR Socorrista |  |
| **2** | Subject heading-MAINSUBJECT | Community Health Workers |  |
| **3** |  | S1 OR S2 | 52251 |
| **4** | abstract | "Primary Health Care" OR "Primary Healthcare” OR “Primary Care” OR “Comprehensive Primary Health Care” OR “Selective Primary Health Care” OR “Vertical Programme” OR "Vertical programmes" OR "Vertical Program" OR "Vertical Programs" OR "Community Health Services" |  |
| **5** | Subject heading-MAINSUBJECT | primary health care OR primary care |  |
| **6** |  | S4 OR S5 | 336833 |
| **7** | abstract | Perception* OR "community perception*" OR "patient perception*" OR perspective OR "Community perspective" OR "patient perspective" OR attitudes OR "attitude to health" OR opinion* OR "opinion of community" OR "opinion of patient" OR feeling* OR "community feelings" OR "feeling of patient" OR experience* OR "community experience" OR " patient experience" OR satisfaction* OR "community satisfaction*" OR "patient satisfaction" OR acceptance* OR "community acceptance*" OR "patient acceptance*" OR "patient acceptance of health care" OR uptake OR utilisation* OR "service utilisation*" OR "community utilisation*" OR "patient utilisation*" OR barrier OR "barrier to health" OR "attitude to health" OR "quality of health care" |  |
| **8** | Subject heading-MAINSUBJECT | "attitude to health" OR "patient acceptance of health care" OR "patient acceptance" OR "community satisfaction OR “patient attitudes” OR “quality of health care” |  |
| **9** |  | S7 OR S8 | 4400445 |
| **10** | abstract | Remote OR "remote area" OR "remote areas" OR "remote region" OR "remote regions" OR rural OR "rural area" OR "rural areas" OR "rural health service" OR "rural health services" OR "Rural Health" OR "rural health center" OR "rural health centers" OR "rural health centre" OR "rural health centres" OR "rural population" OR "rural populations" OR "rural community" OR "rural communities" OR "rural catchments" OR "rural catchment" |  |
| **11** | Subject heading-MAINSUBJECT | Rural OR Rural areas OR rural catchments OR rural communities OR rural health OR rural health care OR rural health services OR rural population OR rural populations |  |
| **12** |  | S10 OR S11 | 1247353 |
| **13** |  | S1 (OR S2) AND S4 (OR S5) AND S7 (OR S8) AND S10 (OR S11) | 561 |
| **14** | After applying Filters | Filters (Duration: 01/01/1990 to 12/6/24, Language: English ) | 558 |

**Search Strategy EBSCOhost CINAHL Ultimate**

| **Search #** | **Field** | **keyword** | **Results** |
| --- | --- | --- | --- |
| **1** | Title | “Health auxiliary” OR “frontline health workers” OR “frontline health worker” OR midwife OR Midwifery OR midwives OR “Birth Attendant” OR Midwives OR “outreach worker” OR “outreach workers” OR “lay health worker” OR “lay health workers” OR promotora OR promotoras OR “village health worker” OR “village health workers” OR “volunteer health worker” OR “volunteer health workers” OR “voluntary health workers” OR “voluntary health worker” OR “community health agent” OR “community health agents” OR “health promoter” OR “health promoters” OR “community health worker” OR “community health workers” OR “community health aide” OR “community health aides” OR “community health nursing” OR “community health nurses” OR “community health nurse” OR “community health officers” OR “community health officer” OR “community health volunteer” OR “community health volunteers” OR “community health distributors” OR “community health distributor” OR “community health surveyors” OR “community health surveyor” OR “community health assistants” OR “community health assistant” OR “community health promoters” OR “community health promoters” OR “community IMCI” OR “community volunteer” OR “community volunteers” OR “health extension workers” OR “health extension worker” OR “village health volunteer” OR “village health volunteers” OR “close-to-community provider” OR “close-to-community providers” OR “community-based practitioner” OR “community-based practitioners” OR “lady Health worker” OR “lady Health workers” OR “barefoot doctor” OR “Community Practitioners” OR “Community Practitioner” OR “community-based practitioners” OR “community-based practitioner” OR “promotoras de salud” OR “agentes de saúde” OR “rural health auxiliaries” OR “traditional birth attendants” OR “traditional birth attendant” OR Activista OR “Agente comunitario de salud” OR “Agente comunitário de saúde” OR Anganwadi OR Animatrice OR “Barangay health worker” OR “Barangay health workers” OR “Basic health worker” OR “Basic health workers” OR Brigadista OR “Colaborador voluntario” OR “Community drug distributor” OR “Community drug distributors” OR “Community health agent” OR “Community health agents” OR “Community health promoter” OR “Community health promoters” OR “Community health representative” OR “Community health representatives” OR “Community health volunteer” OR “Community health volunteers” OR “Community resource person” OR “Female multipurpose health worker” OR “Female multipurpose health worker” OR “Health promoter” OR “Health promoters” OR Kader OR Monitora OR “Mother coordinator” OR “Outreach educator” OR “Outreach educators” OR Promotora OR “Shastho shebika” OR “Shastho karmis” OR Sevika OR “Village health helper” OR “Village drug-kit manager” OR Accompagnateur OR “Accredited Social Health Activist” OR Animator OR ASHA OR “Auxiliary Nurse” OR “Auxiliary Nurse-midwife” OR “Bridge-to-Health Team” OR Behvarz OR “Care Group” OR “Care Groups” OR “Care Group Volunteer” OR “Care Group Volunteers” OR “Community Case Management Worker” OR “Community Case Management Workers” OR “Community Health Agent” OR “Community Health Agents” OR “Community Health Care Provider” OR “Community Health Care Providers” OR “Community HealthCare Provider” OR “Community HealthCare Providers” OR “Community Health Extension Worker” OR “Community Health Extension Workers” OR “Community Health Officer” OR “Community Health Officers” OR “Community Surveillance Volunteer” OR “Community Surveillance Volunteers” OR “Family Health Worker” OR “Family Health Workers” OR “Family Planning Agent” OR “Family Planning Agents” OR “Family Welfare Assistant” OR “Family Welfare Assistants” OR “Female Community Health Volunteer” OR “Female Community Health Volunteers” OR “Health Agent” OR “Health Agents” OR “Health Assistant” OR “Health Assistants” OR “Health Extension Worker” OR “Health Extension Workers” OR “Health Surveillance Assistant” OR “Health Surveillance Assistants” OR “Lead Mother” OR “Malaria Agent” OR “Malaria Agents” OR “Maternal and Child Health Worker” OR “Maternal and Child Health Workers” OR “Mobile Clinic Team” OR “Mobile Clinic Teams” OR “Nutrition Agent” OR “Nutrition Agents” OR “Nutrition Counselor” OR “Nutrition Counselors” OR “Peer Educator” OR “Peer Educators” OR “Shasthya Shebika” OR Socorrista |  |
| **2** | abstract | “Health auxiliary” OR “frontline health workers” OR “frontline health worker” OR midwife OR Midwifery OR midwives OR “Birth Attendant” OR Midwives OR “outreach worker” OR “outreach workers” OR “lay health worker” OR “lay health workers” OR promotora OR promotoras OR “village health worker” OR “village health workers” OR “volunteer health worker” OR “volunteer health workers” OR “voluntary health workers” OR “voluntary health worker” OR “community health agent” OR “community health agents” OR “health promoter” OR “health promoters” OR “community health worker” OR “community health workers” OR “community health aide” OR “community health aides” OR “community health nursing” OR “community health nurses” OR “community health nurse” OR “community health officers” OR “community health officer” OR “community health volunteer” OR “community health volunteers” OR “community health distributors” OR “community health distributor” OR “community health surveyors” OR “community health surveyor” OR “community health assistants” OR “community health assistant” OR “community health promoters” OR “community health promoters” OR “community IMCI” OR “community volunteer” OR “community volunteers” OR “health extension workers” OR “health extension worker” OR “village health volunteer” OR “village health volunteers” OR “close-to-community provider” OR “close-to-community providers” OR “community-based practitioner” OR “community-based practitioners” OR “lady Health worker” OR “lady Health workers” OR “barefoot doctor” OR “Community Practitioners” OR “Community Practitioner” OR “community-based practitioners” OR “community-based practitioner” OR “promotoras de salud” OR “agentes de saúde” OR “rural health auxiliaries” OR “traditional birth attendants” OR “traditional birth attendant” OR Activista OR “Agente comunitario de salud” OR “Agente comunitário de saúde” OR Anganwadi OR Animatrice OR “Barangay health worker” OR “Barangay health workers” OR “Basic health worker” OR “Basic health workers” OR Brigadista OR “Colaborador voluntario” OR “Community drug distributor” OR “Community drug distributors” OR “Community health agent” OR “Community health agents” OR “Community health promoter” OR “Community health promoters” OR “Community health representative” OR “Community health representatives” OR “Community health volunteer” OR “Community health volunteers” OR “Community resource person” OR “Female multipurpose health worker” OR “Female multipurpose health worker” OR “Health promoter” OR “Health promoters” OR Kader OR Monitora OR “Mother coordinator” OR “Outreach educator” OR “Outreach educators” OR Promotora OR “Shastho shebika” OR “Shastho karmis” OR Sevika OR “Village health helper” OR “Village drug-kit manager” OR Accompagnateur OR “Accredited Social Health Activist” OR Animator OR ASHA OR “Auxiliary Nurse” OR “Auxiliary Nurse-midwife” OR “Bridge-to-Health Team” OR Behvarz OR “Care Group” OR “Care Groups” OR “Care Group Volunteer” OR “Care Group Volunteers” OR “Community Case Management Worker” OR “Community Case Management Workers” OR “Community Health Agent” OR “Community Health Agents” OR “Community Health Care Provider” OR “Community Health Care Providers” OR “Community HealthCare Provider” OR “Community HealthCare Providers” OR “Community Health Extension Worker” OR “Community Health Extension Workers” OR “Community Health Officer” OR “Community Health Officers” OR “Community Surveillance Volunteer” OR “Community Surveillance Volunteers” OR “Family Health Worker” OR “Family Health Workers” OR “Family Planning Agent” OR “Family Planning Agents” OR “Family Welfare Assistant” OR “Family Welfare Assistants” OR “Female Community Health Volunteer” OR “Female Community Health Volunteers” OR “Health Agent” OR “Health Agents” OR “Health Assistant” OR “Health Assistants” OR “Health Extension Worker” OR “Health Extension Workers” OR “Health Surveillance Assistant” OR “Health Surveillance Assistants” OR “Lead Mother” OR “Malaria Agent” OR “Malaria Agents” OR “Maternal and Child Health Worker” OR “Maternal and Child Health Workers” OR “Mobile Clinic Team” OR “Mobile Clinic Teams” OR “Nutrition Agent” OR “Nutrition Agents” OR “Nutrition Counselor” OR “Nutrition Counselors” OR “Peer Educator” OR “Peer Educators” OR “Shasthya Shebika” OR Socorrista |  |
| **3** | MH Exact Subject Heading | Community Health Workers |  |
| **4** |  | S1 OR S2 OR S3 | 53529 |
| **5** | Title | "Primary Health Care" OR "Primary Healthcare” OR “Primary Care” OR “Comprehensive Primary Health Care” OR “Selective Primary Health Care” OR “Vertical Programme” OR "Vertical programmes" OR "Vertical Program" OR "Vertical Programs" OR "Community Health Services" |  |
| **6** | Abstract | "Primary Health Care" OR "Primary Healthcare” OR “Primary Care” OR “Comprehensive Primary Health Care” OR “Selective Primary Health Care” OR “Vertical Programme” OR "Vertical programmes" OR "Vertical Program" OR "Vertical Programs" OR "Community Health Services" |  |
| **7** | MH Exact Subject Heading | Primary Health Care OR Community Health Services |  |
| **8** |  | S5 OR S6 OR S7 | 152506 |
| **9** | Title | Perception* OR "community perception*" OR "patient perception*" OR perspective OR "Community perspective" OR "patient perspective" OR attitudes OR "attitude to health" OR opinion* OR "opinion of community" OR "opinion of patient" OR feeling* OR "community feelings" OR "feeling of patient" OR experience* OR "community experience" OR " patient experience" OR satisfaction* OR "community satisfaction*" OR "patient satisfaction" OR acceptance* OR "community acceptance*" OR "patient acceptance*" OR "patient acceptance of health care" OR uptake OR utilisation* OR "service utilisation*" OR "community utilisation*" OR "patient utilisation*" OR barrier OR "barrier to health" OR "attitude to health" OR "quality of health care" |  |
| **10** | Abstract | Perception* OR "community perception*" OR "patient perception*" OR perspective OR "Community perspective" OR "patient perspective" OR attitudes OR "attitude to health" OR opinion* OR "opinion of community" OR "opinion of patient" OR feeling* OR "community feelings" OR "feeling of patient" OR experience* OR "community experience" OR " patient experience" OR satisfaction* OR "community satisfaction*" OR "patient satisfaction" OR acceptance* OR "community acceptance*" OR "patient acceptance*" OR "patient acceptance of health care" OR uptake OR utilisation* OR "service utilisation*" OR "community utilisation*" OR "patient utilisation*" OR barrier OR "barrier to health" OR "attitude to health" OR "quality of health care" |  |
| **11** | MH Exact Subject Heading | "Attitude to health" OR “Patient attitudes” OR “Patient satisfaction” OR “Quality of health care” |  |
| **12** |  | S9 OR S10 OR S11 | 1222617 |
| **13** | Title | Remote OR "remote area" OR "remote areas" OR "remote region" OR "remote regions" OR rural OR "rural area" OR "rural areas" OR "rural health service" OR "rural health services" OR "Rural Health" OR "rural health center" OR "rural health centers" OR "rural health centre" OR "rural health centres" OR "rural population" OR "rural populations" OR "rural community" OR "rural communities" OR "rural catchments" OR "rural catchment" |  |
| **14** | Abstract | Remote OR "remote area" OR "remote areas" OR "remote region" OR "remote regions" OR rural OR "rural area" OR "rural areas" OR "rural health service" OR "rural health services" OR "Rural Health" OR "rural health center" OR "rural health centers" OR "rural health centre" OR "rural health centres" OR "rural population" OR "rural populations" OR "rural community" OR "rural communities" OR "rural catchments" OR "rural catchment" |  |
| **15** | MH Exact Subject Heading | rural population OR rural areas OR rural health services OR rural health OR rural health centres |  |
| **16** |  | S13 OR S14 OR S15 | 103005 |
| **17** |  | S1 (OR S2 OR S3) AND S5 (OR S6 OR S7) AND S9 (OR S10 OR S11) AND S13 (OR S14 OR S15) | 366 |
| **18** | After applying filters | Filters (Duration: 1990-2024, Language: English) | 360 |

**Search Strategy for APA PsycINFO**

| **Search**  **#** | **Field** | **Keyword** | **Results** |
| --- | --- | --- | --- |
| **1** | Title | “Health auxiliary” OR “frontline health workers” OR “frontline health worker” OR midwife OR Midwifery OR midwives OR “Birth Attendant” OR Midwives OR “outreach worker” OR “outreach workers” OR “lay health worker” OR “lay health workers” OR promotora OR promotoras OR “village health worker” OR “village health workers” OR “volunteer health worker” OR “volunteer health workers” OR “voluntary health workers” OR “voluntary health worker” OR “community health agent” OR “community health agents” OR “health promoter” OR “health promoters” OR “community health worker” OR “community health workers” OR “community health aide” OR “community health aides” OR “community health nursing” OR “community health nurses” OR “community health nurse” OR “community health officers” OR “community health officer” OR “community health volunteer” OR “community health volunteers” OR “community health distributors” OR “community health distributor” OR “community health surveyors” OR “community health surveyor” OR “community health assistants” OR “community health assistant” OR “community health promoters” OR “community health promoters” OR “community IMCI” OR “community volunteer” OR “community volunteers” OR “health extension workers” OR “health extension worker” OR “village health volunteer” OR “village health volunteers” OR “close-to-community provider” OR “close-to-community providers” OR “community-based practitioner” OR “community-based practitioners” OR “lady Health worker” OR “lady Health workers” OR “barefoot doctor” OR “Community Practitioners” OR “Community Practitioner” OR “community-based practitioners” OR “community-based practitioner” OR “promotoras de salud” OR “agentes de saúde” OR “rural health auxiliaries” OR “traditional birth attendants” OR “traditional birth attendant” OR Activista OR “Agente comunitario de salud” OR “Agente comunitário de saúde” OR Anganwadi OR Animatrice OR “Barangay health worker” OR “Barangay health workers” OR “Basic health worker” OR “Basic health workers” OR Brigadista OR “Colaborador voluntario” OR “Community drug distributor” OR “Community drug distributors” OR “Community health agent” OR “Community health agents” OR “Community health promoter” OR “Community health promoters” OR “Community health representative” OR “Community health representatives” OR “Community health volunteer” OR “Community health volunteers” OR “Community resource person” OR “Female multipurpose health worker” OR “Female multipurpose health worker” OR “Health promoter” OR “Health promoters” OR Kader OR Monitora OR “Mother coordinator” OR “Outreach educator” OR “Outreach educators” OR Promotora OR “Shastho shebika” OR “Shastho karmis” OR Sevika OR “Village health helper” OR “Village drug-kit manager” OR Accompagnateur OR “Accredited Social Health Activist” OR Animator OR ASHA OR “Auxiliary Nurse” OR “Auxiliary Nurse-midwife” OR “Bridge-to-Health Team” OR Behvarz OR “Care Group” OR “Care Groups” OR “Care Group Volunteer” OR “Care Group Volunteers” OR “Community Case Management Worker” OR “Community Case Management Workers” OR “Community Health Agent” OR “Community Health Agents” OR “Community Health Care Provider” OR “Community Health Care Providers” OR “Community HealthCare Provider” OR “Community HealthCare Providers” OR “Community Health Extension Worker” OR “Community Health Extension Workers” OR “Community Health Officer” OR “Community Health Officers” OR “Community Surveillance Volunteer” OR “Community Surveillance Volunteers” OR “Family Health Worker” OR “Family Health Workers” OR “Family Planning Agent” OR “Family Planning Agents” OR “Family Welfare Assistant” OR “Family Welfare Assistants” OR “Female Community Health Volunteer” OR “Female Community Health Volunteers” OR “Health Agent” OR “Health Agents” OR “Health Assistant” OR “Health Assistants” OR “Health Extension Worker” OR “Health Extension Workers” OR “Health Surveillance Assistant” OR “Health Surveillance Assistants” OR “Lead Mother” OR “Malaria Agent” OR “Malaria Agents” OR “Maternal and Child  Health Worker” OR “Maternal and Child Health Workers” OR “Mobile Clinic Team” OR “Mobile Clinic Teams” OR “Nutrition Agent” OR “Nutrition Agents” OR “Nutrition Counselor” OR “Nutrition Counselors” OR “Peer Educator” OR “Peer Educators” OR “Shasthya Shebika” OR Socorrista |  |
| **2** | Abstract | “Health auxiliary” OR “frontline health workers” OR “frontline health worker” OR midwife OR Midwifery OR midwives OR “Birth Attendant” OR Midwives OR “outreach worker” OR “outreach workers” OR “lay health worker” OR “lay health workers” OR promotora OR promotoras OR “village health worker” OR “village health workers” OR “volunteer health worker” OR “volunteer health workers” OR “voluntary health workers” OR “voluntary health worker” OR “community health agent” OR “community health agents” OR “health promoter” OR “health promoters” OR “community health worker” OR “community health workers” OR “community health aide” OR “community health aides” OR “community health nursing” OR “community health nurses” OR “community health nurse” OR “community health officers” OR “community health officer” OR “community health volunteer” OR “community health volunteers” OR “community health distributors” OR “community health distributor” OR “community health surveyors” OR “community health surveyor” OR “community health assistants” OR “community health assistant” OR “community health promoters” OR “community health promoters” OR “community IMCI” OR “community volunteer” OR “community volunteers” OR “health extension workers” OR “health extension worker” OR “village health volunteer” OR “village health volunteers” OR “close-to-community provider” OR “close-to-community providers” OR “community-based practitioner” OR “community-based practitioners” OR “lady Health worker” OR “lady Health workers” OR “barefoot doctor” OR “Community Practitioners” OR “Community Practitioner” OR “community-based practitioners” OR “community-based practitioner” OR “promotoras de salud” OR “agentes de saúde” OR “rural health auxiliaries” OR “traditional birth attendants” OR “traditional birth attendant” OR Activista OR “Agente comunitario de salud” OR “Agente comunitário de saúde” OR Anganwadi OR Animatrice OR “Barangay health worker” OR “Barangay health workers” OR “Basic health worker” OR “Basic health workers” OR Brigadista OR “Colaborador voluntario” OR “Community drug distributor” OR “Community drug distributors” OR “Community health agent” OR “Community health agents” OR “Community health promoter” OR “Community health promoters” OR “Community health representative” OR “Community health representatives” OR “Community health volunteer” OR “Community health volunteers” OR “Community resource person” OR “Female multipurpose health worker” OR “Female multipurpose health worker” OR “Health promoter” OR “Health promoters” OR Kader OR Monitora OR “Mother coordinator” OR “Outreach educator” OR “Outreach educators” OR Promotora OR “Shastho shebika” OR “Shastho karmis” OR Sevika OR “Village health helper” OR “Village drug-kit manager” OR Accompagnateur OR “Accredited Social Health Activist” OR Animator OR ASHA OR “Auxiliary Nurse” OR “Auxiliary Nurse-midwife” OR “Bridge-to-Health Team” OR Behvarz OR “Care Group” OR “Care Groups” OR “Care Group Volunteer” OR “Care Group Volunteers” OR “Community Case Management Worker” OR “Community Case Management Workers” OR “Community Health Agent” OR “Community Health Agents” OR “Community Health Care Provider” OR “Community Health Care Providers” OR “Community HealthCare Provider” OR “Community HealthCare Providers” OR “Community Health Extension Worker” OR “Community Health Extension Workers” OR “Community Health Officer” OR “Community Health Officers” OR “Community Surveillance Volunteer” OR “Community Surveillance Volunteers” OR “Family Health Worker” OR “Family Health Workers” OR “Family Planning Agent” OR “Family Planning Agents” OR “Family Welfare Assistant” OR “Family Welfare Assistants” OR “Female Community Health Volunteer” OR “Female Community Health Volunteers” OR “Health Agent” OR “Health Agents” OR “Health Assistant” OR “Health Assistants” OR “Health Extension Worker” OR “Health Extension Workers” OR “Health Surveillance Assistant” OR “Health Surveillance Assistants” OR “Lead Mother” OR “Malaria Agent” OR “Malaria Agents” OR “Maternal and Child Health Worker” OR “Maternal and Child Health Workers” OR “Mobile Clinic Team” OR “Mobile Clinic Teams” OR “Nutrition Agent” OR “Nutrition Agents” OR “Nutrition Counselor” OR “Nutrition Counselors” OR “Peer Educator” OR “Peer Educators” OR “Shasthya Shebika” OR Socorrista |  |
| **3** | MeSH | (No MeSH term) |  |
| **4** |  | S1 OR S2 | 10419 |
| **5** | Title | "Primary Health Care" OR "Primary Healthcare” OR “Primary Care” OR “Comprehensive Primary Health Care” OR “Selective Primary Health Care” OR “Vertical Programme” OR "Vertical programmes" OR "Vertical Program" OR "Vertical Programs" OR "Community Health Services" |  |
| **6** | Abstract | "Primary Health Care" OR "Primary Healthcare” OR “Primary Care” OR “Comprehensive Primary Health Care” OR “Selective Primary Health Care” OR “Vertical Programme” OR "Vertical programmes" OR "Vertical Program" OR "Vertical Programs" OR "Community Health Services" |  |
| **7** | MeSH | primary health care |  |
| **8** |  | S5 OR S6 OR S7 | 42635 |
| **9** | Title | Perception* OR "community perception*" OR "patient perception*" OR perspective OR "Community perspective" OR "patient perspective" OR attitudes OR "attitude to health" OR opinion* OR "opinion of community" OR "opinion of patient" OR feeling* OR "community feelings" OR "feeling of patient" OR experience* OR "community experience" OR " patient experience" OR satisfaction* OR "community satisfaction*" OR "patient satisfaction" OR acceptance* OR "community acceptance*" OR "patient acceptance*" OR "patient acceptance of health care" OR uptake OR utilisation* OR "service utilisation*" OR "community utilisation*" OR "patient utilisation*" OR barrier OR "barrier to health" OR "attitude to health" OR "quality of health care" |  |
| **10** | Abstract | Perception* OR "community perception*" OR "patient perception*" OR perspective OR "Community perspective" OR "patient perspective" OR attitudes OR "attitude to health" OR opinion* OR "opinion of community" OR "opinion of patient" OR feeling* OR "community feelings" OR "feeling of patient" OR experience* OR "community experience" OR " patient experience" OR satisfaction* OR "community satisfaction*" OR "patient satisfaction" OR acceptance* OR "community acceptance*" OR "patient acceptance*" OR "patient acceptance of health care" OR uptake OR utilisation* OR "service utilisation*" OR "community utilisation*" OR "patient utilisation*" OR barrier OR "barrier to health" OR "attitude to health" OR "quality of health care" |  |
| **11** | MeSH | Community attitudes OR Client attitudes OR client satisfaction OR quality of care OR Health care utilisation OR community involvement |  |
| **12** |  | S9 OR S10 ORS11 | 1590828 |
| **13** | Title | Remote OR "remote area" OR "remote areas" OR "remote region" OR "remote regions" OR rural OR "rural area" OR "rural areas" OR "rural health service" OR "rural health services" OR "Rural Health" OR "rural health center" OR "rural health centers" OR "rural health centre" OR "rural health centres" OR "rural population" OR "rural populations" OR "rural community" OR "rural communities" OR "rural catchments" OR "rural catchment" |  |
| **14** | Abstract | Remote OR "remote area" OR "remote areas" OR "remote region" OR "remote regions" OR rural OR "rural area" OR "rural areas" OR "rural health service" OR "rural health services" OR "Rural Health" OR "rural health center" OR "rural health centers" OR "rural health centre" OR "rural health centres" OR "rural population" OR "rural populations" OR "rural community" OR "rural communities" OR "rural catchments" OR "rural catchment" |  |
| **15** | MeSH | rural health |  |
| **16** |  | S13 OR S14 OR S15 | 68806 |
| **17** |  | S1 (OR S2) AND S4 (OR S5) AND S7 (OR S8) AND S10 (OR S11) | 48 |
| **18** | After applying filters | Filters (Duration: 1990-2024, Language: English) | 47 |
