# Supplementary material for: Factors Influencing Community Members' Perception of Primary Health Care Services Delivered by Community Health Workers in Rural Areas: A Systematic Review
Source: Aust J Rural Health. 2026 Jan 19;34(1):e70142. doi: 10.1111/ajr.70142 (PMC12921660; doi:10.1111/ajr.70142)
Supplement: Supplementary file 2 — Supporting Information S2. [file AJR-34-0-s002.docx]

**Supplementary Material 2**

**Mixed Method Appraisal Tool (MMAT) 2018**

**MMAT Score**

| **QUALITATIVE STUDY** | | | | | | | |
| --- | --- | --- | --- | --- | --- | --- | --- |
| **Study characteristics** | **Screening questions** | | **MMAT criteria** | | | | |
|  | Are there clear research questions? | Do the collected data allow to address the research questions? | Is the qualitative approach appropriate to answer the research question? | Are the qualitative data collection methods adequate to address the research question? | Are the findings adequately derived from the data? | Is the interpretation of results sufficiently substantiated by data? | Is there coherence between qualitative data sources, collection, analysis and interpretation? |
| Walker et al. 2020  Eswatani | 1 | 1 | 1 | 1 | 1 | 1 | 1 |
| Adamu 2012  Ethiopia | 1 | 1 | 1 | 1 | 1 | 1 | 1 |
| Agraw et al. 2007  Ethiopia (Score for the qualitative part of the study) | 1 | 1 | 1 | 1 | 1 | 1 | 1 |
| Shaw et al. 2017  Ethiopia | 1 | 1 | 1 | 1 | 1 | 1 | 1 |
| Yigru et al. 2020  Ethiopia | 1 | 1 | 1 | 1 | 1 | 1 | 1 |
| Baiden et al. 2007  Ghana (Scores for Qualitative Part of the study) | 1 | 1 | 1 | 1 | 1 | 1 | 1 |
| Stephens et al. 2020  Ghana | 1 | 1 | 1 | 1 | 1 | 1 | 1 |
| Akilan et al. 2014  India | 1 | 1 | 1 | 1 | 1 | 1 | 1 |
| Blanchard 2024  India | 1 | 1 | 1 | 1 | 1 | 1 | 1 |
| Kamau 2020  Kenya | 1 | 1 | 1 | 1 | 1 | 1 | 1 |
| Kibel et al. 2020  Kenya | 1 | 1 | 1 | 1 | 1 | 1 | 1 |
| Ndambo 2024  Malawi | 1 | 1 | 1 | 1 | 1 | 1 | 1 |
| Watt et al. 2016  Myanmar | 1 | 1 | 1 | 1 | 1 | 1 | 1 |
| Ratima et al. 1999  New Zealand | 1 | 1 | 1 | 1 | 1 | 1 | 1 |
| Grant et al. 2017  South Africa | 1 | 1 | 1 | 1 | 1 | 1 | 1 |
| Wilford et al. 2018  South Africa | 1 | 1 | 1 | 1 | 1 | 1 | 1 |
| Rafiq et al. 2019  Tanzania | 1 | 1 | 1 | 1 | 1 | 1 | 1 |
| York et al. 2014  Tanzania (Score for the qualitative part of the study) | 1 | 1 | 1 | 1 | 1 | 1 | 1 |
| Batte et al. 2021  Uganda | 1 | 1 | 1 | 1 | 1 | 1 | 1 |
| Buchner et al. 2014  Uganda | 1 | 1 | 1 | 1 | 1 | 1 | 1 |
| Waiswa et al. 2008  Uganda | 1 | 1 | 1 | 1 | 1 | 1 | 1 |

| **QUANTITATIVE DESCRIPTIVE STUDIES** | | | | | | | |
| --- | --- | --- | --- | --- | --- | --- | --- |
| **Study characteristics** | **Screening questions** | | **MMAT criteria** | | | | |
|  | Are there clear research questions? | Do the collected data allow to address the research questions? | Is the sampling strategy relevant to address the research question? | Is the sample representative of the target population? | Are the measurements appropriate? | Is the risk of nonresponse bias low? | Is the statistical analysis appropriate to answer the research question? |
| Atkinson and Haran, 2004  Brazil | 1 | 1 | 1 | 1 | 1 | 1 | 1 |
| Agraw et al. 2007  Ethiopia (Scores for the quantitative part of the study) | 1 | 1 | 1 | 1 | 1 | 1 | 1 |
| Madhanyie et al.2012  Ethiopia | 1 | 1 | 1 | 1 | 1 | 1 | 1 |
| Shaw et al. 2015  Ethiopia | 1 | 1 | 1 | 1 | 1 | 1 | 1 |
| Tesfaye et al. 2014  Ethiopia | 1 | 1 | 1 | 1 | 1 | 1 | 1 |
| Baiden et al. 2007  Ghana (Score for the quantitative part of the study) | 1 | 1 | 1 | 1 | 1 | Can’t tell | 1 |
| Juma et al. 2015  Kenya | 1 | 1 | 1 | 1 | 1 | 1 | 1 |
| Rogers et al. 2022  Kenya | 1 | 1 | 1 | 1 | 1 | 1 | 1 |
| Okereke et al. 2020  Nigeria | 1 | 1 | 1 | 1 | 1 | 1 | 1 |
| York et al. 2014  Tanzania (Score for the quantitative part of the study) | 1 | 1 | 1 | 1 | 1 | 1 | 1 |

| **MIXED METHODS STUDIES** | | | | | | | |
| --- | --- | --- | --- | --- | --- | --- | --- |
| **Study characteristics** | **Screening questions** | | **MMAT criteria** | | | | |
|  | Are there clear research questions? | Do the collected data allow to address the research questions? | Is there an adequate rationale for using a mixed methods design to address the research question? | Are the different components of the study effectively integrated to answer the research question? | Are the outputs of the integration of qualitative and quantitative components adequately interpreted? | Are divergences and inconsistencies between quantitative and qualitative results adequately addressed? | Do the different components of the study adhere to the quality criteria of each tradition of the methods involved? |
| Puett et al. 2013  Bangladesh | 1 | 1 | 1 | 1 | 0 | 1 | 1 |
| Henning et al. 2020  Zambia | 1 | 1 | 0 | 1 | 1 | 0 | 1 |
